# The Highly Conserved Bacterial RNase YbeY Is Essential in *Vibrio cholerae*, Playing a Critical Role in Virulence, Stress Regulation, and RNA Processing

**DOI:** 10.1371/journal.ppat.1004175

**Published:** 2014-06-05

**Authors:** Maarten Vercruysse, Caroline Köhrer, Bryan W. Davies, Markus F. F. Arnold, John J. Mekalanos, Uttam L. RajBhandary, Graham C. Walker

**Affiliations:** 1 Department of Biology, Massachusetts Institute of Technology, Cambridge, Massachusetts, United States of America; 2 Department of Molecular Biosciences, The University of Texas at Austin, Austin, Texas, United States of America; 3 Department of Microbiology and Immunobiology, Harvard Medical School, Boston, Massachussets, United States of America; University of Texas San Antonio, United States of America

## Abstract

YbeY, a highly conserved protein, is an RNase in *E. coli* and plays key roles in both processing of the critical 3′ end of 16 S rRNA and in 70 S ribosome quality control under stress. These central roles account for YbeY's inclusion in the postulated minimal bacterial genome. However, YbeY is not essential in *E. coli* although loss of *ybeY* severely sensitizes it to multiple physiological stresses. Here, we show that YbeY is an essential endoribonuclease in *Vibrio cholerae* and is crucial for virulence, stress regulation, RNA processing and ribosome quality control, and is part of a core set of RNases essential in most representative pathogens. To understand its function, we analyzed the rRNA and ribosome profiles of a *V. cholerae* strain partially depleted for YbeY and other RNase mutants associated with 16 S rRNA processing; our results demonstrate that YbeY is also crucial for 16 S rRNA 3′ end maturation in *V. cholerae* and that its depletion impedes subunit assembly into 70 S ribosomes. YbeY's importance to *V. cholerae* pathogenesis was demonstrated by the complete loss of mice colonization and biofilm formation, reduced cholera toxin production, and altered expression levels of virulence-associated small RNAs of a *V. cholerae* strain partially depleted for YbeY. Notably, the *ybeY* genes of several distantly related pathogens can fully complement an *E. coli ΔybeY* strain under various stress conditions, demonstrating the high conservation of YbeY's activity in stress regulation. Taken together, this work provides the first comprehensive exploration of YbeY's physiological role in a human pathogen, showing its conserved function across species in essential cellular processes.

## Author Summary

Bacteria adapt and survive unfavorable environments by quickly changing their gene expression and physiology, for example as pathogens do during infection of host cells. Gene expression is often determined by RNA turnover, a balance between transcription and RNA decay carried out by multiple RNases. The recently identified RNase YbeY was shown in *E. coli* to participate in rRNA maturation and 70 S ribosome quality control, however YbeY's roles in other organisms and the extent of functional conservation is unknown. Here, we show that YbeY is an essential RNase in the pathogen *Vibrio cholerae*, critical for cell fitness and general stress tolerance. We demonstrate that YbeY is crucial for 16 S rRNA 3′ end maturation, assembly of functional 70 S ribosomes and ribosome quality control. Moreover, YbeY regulates virulence-associated small RNAs and its depletion leads to an overall reduction in pathogenesis, exemplified by significantly decreased biofilm formation, mouse colonization and cholera toxin production. We also show that YbeY belongs to a minimal core set of RNases essential in most representative pathogens. The multifaceted roles of YbeY in several essential cellular processes and its highly conserved function across bacterial species, suggest that YbeY could be an attractive new antimicrobial target.

## Introduction

To thrive in diverse and changing environments, bacteria have to quickly sense and respond to a broad range of stimuli and signals. The ability to adapt to environmental changes is particularly important for pathogens during invasion of the host. Bacterial adaptation is often mediated through modulation of gene expression at the post-transcriptional level. A common mechanism for post-transcriptional control of gene expression is global mRNA stability, which in term is regulated by ribonucleases (RNases) that act either directly on their target mRNAs or in conjunction with regulatory RNAs [1]–[6].

RNases, which can be divided into endo- and exonucleases according to their substrate specificities, play crucial roles in bacterial pathogenesis by regulating the expression of many virulence factors. In uropathogenic *Escherichia coli*, the endoribonuclease RNase E controls expression of Pap pili required for attachment to the kidney by selectively processing the bicistronic *papBA* mRNA [7]. In *Stapylococcus aureus*, the endoribonuclease RNase III mediates the degradation of duplexes of RNAIII with mRNAs coding for early virulence factors, whereas the exoribonuclease PNPase is involved in the cold stress response and global mRNA turnover [3], [8]. In Gram-positive bacteria, RNase Y is the functional equivalent of RNase E in *E. coli* and affects virulence of *S. aureus* and *Streptococcus pyogenes* in silkworm and mouse models [3].

Small RNAs (sRNAs) that control gene expression are perfectly suited to initiate rapid regulatory circuits, as they do not require translation and can be turned over quickly by various RNases [5], [9]. For example, in *Vibrio cholerae*, several sRNAs have been identified with a role in pathogenesis, such as the quorum-sensing sRNAs Qrr1–4 and the ToxT activated sRNAs TarA and TarB [10], [11]. In *S. aureus*, RNAIII acts as both an activator and repressor of early virulence factors, and 6S RNA of *Legionella pneumophila* is crucial for replication in host cells [12], [13]. The function of regulatory sRNAs often relies on their interactions with accessory proteins, e.g. the RNA chaperone Hfq for stabilization of the sRNA/target mRNA interaction and various RNases for specific degradation of sRNAs and their target mRNAs [2], [5].

One of the most recently identified RNases is YbeY, a highly conserved member of the UPF0054 protein family. It is found in almost all sequenced bacteria and is part of the postulated minimal bacterial gene set, encompassing 206 genes [14], [15]. YbeY is not essential in *E. coli* and *Sinorhizobium meliloti* but its loss results in severe sensitivity to various stresses [14], [16]. Studies of YbeY in *E. coli* have demonstrated that YbeY is a single-strand specific endoribonuclease with multiple physiological functions [16]–[19]. Deletion of *ybeY* in *E. coli* affects maturation of all three ribosomal RNAs (rRNA), in particular 16 S rRNA. The deficiencies in rRNA processing caused by loss of YbeY function are augmented by additional mutations in *rnc, rnr*, and *pnp*, encoding RNase III, RNase R, and PNPase, respectively. Correct maturation of the 3′ end of 16 S rRNA is essentially lost in Δ*ybeY* Δ*rnr* and Δ*ybeY* Δ*pnp* double mutants, making YbeY the first reported endoribonuclease shown to be involved in this critically important processing step [16]. 17 S rRNA and 16 S* rRNA, a truncated 16 S rRNA species, also strongly accumulate in a Δ*ybeY* strain after heat stress, consistent with YbeY's role in the heat-shock response in *E. coli* [18], [20]. The efficiency and accuracy of translation is significantly lower in a Δ*ybeY* strain, most likely due to maturation defects in the 30 S ribosomal subunits [16], [19]. Protein mistranslation can be toxic to cells and therefore needs to be prevented. Interestingly, YbeY acting together with RNase R can remove defective 70 S ribosomes in a recently discovered mechanism of late-stage ribosome quality control in *E. coli* [18]. The latter process specifically targets fully assembled 70 S ribosomes containing immature or defective 30 S subunits [18]. An additional link to RNA metabolism is YbeY's role in sRNA-mediated regulation of gene expression. Deletion of *ybeY* in *S. meliloti* produces a phenotype very similar to that of an *hfq* mutant, and several sRNAs were identified that potentially control expression of YbeY- and Hfq-dependent genes [21]. In view of YbeY's multiple functions in bacterial RNA metabolism and the importance of RNases in pathogens, we decided to study YbeY's physiological roles in a representative human pathogen.

*Vibrio cholerae* is the causative agent of cholera, a potentially lethal diarrheal disease that remains an important global health problem, having caused seven pandemics since 1817 [22]–[24]. This highly motile γ-Proteobacterium lives in both aquatic and mammalian intestinal environments. Its transmission occurs through contaminated water in regions with poor sanitation or by ingestion of contaminated shellfish, algae and aquatic plants, which are *Vibrio*'s ecological hosts [25]. Most pathogenesis genes of *V. cholerae* 7^th^ pandemic O1 El Tor biotype strain N16961 are found on the larger of the two chromosomes including toxins, surface antigens and adhesins [26]. The two major virulence factors are the cholera toxin (CT), encoded by *ctxAB* on the CTXφ prophage [27], and the toxin co-regulated pilus (TCP), encoded by the *tcp* operon on the TCP pathogenicity island [28]. Following colonization of the small intestine, the A subunit of the AB_5_-toxin complex is taken into the intestinal epithelial cell via endocytosis leading to an increase of the host cell's cAMP levels by constitutive activation of adenylate cyclase and causing an efflux of chloride ions and water.

Here, we show that YbeY is an essential gene in *V. cholerae* and that normal levels of its expression are crucial for virulence. A phylogenetic comparison of the essentiality of major RNases shows that YbeY is part of a small set of RNases essential in most representative pathogens. To understand its functional characteristics, we analyzed the rRNA and ribosome profiles of a *V. cholerae* strain after YbeY depletion, observing gradual accumulation of 16 S rRNA precursors and free ribosomal subunits. Compared to the 16 S rRNA processing RNase G [29] and the exonuclease RNase R, also shown to be involved in 16 S rRNA processing [16], [30], only the loss of YbeY led to severe defects in 16 S rRNA 3′ end maturation and 70 S ribosome assembly. In the case of the multifunctional RNase E [31], the gene could not be deleted because it is essential, but a strain encoding RNase E:ΔCTD, in which the carboxy-terminal domain is disrupted), had no effect on 16 S processing. YbeY is also crucial for the virulence of *V. cholerae*, since a *ΔybeY* depletion strain that expresses reduced levels of YbeY fails to colonize infant mice intestines and its biofilm and CT production are significantly reduced compared to wild-type (Wt). In addition, we experimentally identified several YbeY- and RNase E:ΔCTD-dependent sRNAs associated with *V. cholerae* virulence. Strikingly, *ybeY* genes from four distantly related pathogens can fully complement an *E. coli ΔybeY* strain, demonstrating the high conservation of YbeY activity across species.

## Results

### YbeY is part of a core set of RNases essential in most representative pathogens

To date, YbeY's physiological roles have been studied in *E. coli* [16]–[19], [32] and *S. meliloti* [14], [21]. Its multifaceted functions in rRNA and sRNA metabolism make YbeY a key player among RNases. To gain further insight into the role of YbeY within the network of bacterial RNases, we carried out a comprehensive phylogenetic analysis of RNases in a selection of bacteria with a focus on human pathogens, assessing (i) the presence or absence of major RNases and (ii) their essentiality.

**Figure 1:**
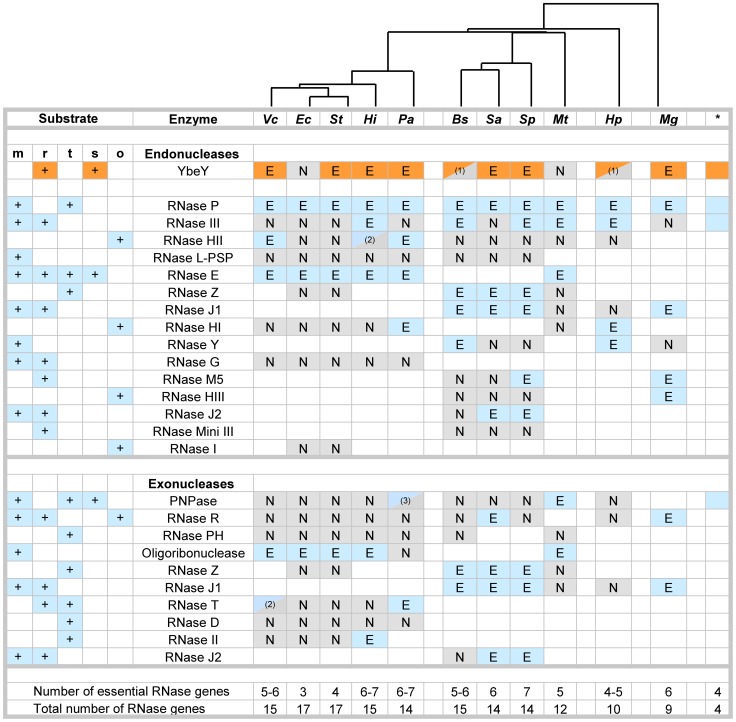
Essential RNases in human pathogens. Compilation of major RNases in *V. cholerae* (*Vc*), *E. coli* (*Ec*), *S. typhi* (*St*), *H. influenzae* (*Hi*), *P. aeruginosa* (*Pa*), *B. subtilis* (*Bs*), *S. aureus* (*Sa*), *S. pneumoniae* (*Sp*), *M. tuberculosis* (*Mt*), *H. pylori* (*Hp*), and *M. genitalium* (*Mg*). The asterisk indicates RNases present in the minimal bacterial genome set [15]. RNases with dual endo- and exonuclease functions are listed twice, but are counted only once to assess the total number of genes. Blank squares indicate that no homolog for the respective RNase could be identified. The designation of RNases as putatively essential (E) and non-essential (N) is based on the availability of deletion or transposon mutants according to Materials and Methods. ^(1)^ Current databases list these RNases as non-essential; follow-up experiments suggest these RNases could by essential (personal communication; BW Davies, LA Simmons, Y Furuta). ^(2)^ RNases were listed as essential in one database and non-essential in a second database. ^(3)^ Only mutants with transposon insertions in the last 10% of the gene are available. The key roles of RNases in mRNA (m), rRNA (r), tRNA (t) and sRNA (s) metabolism are indicated; other functions (o). The phylogenetic tree on top is based on alignment of various YbeY protein sequences using Geneious software.

Figure 1 summarizes the results of our analysis for the main endo- and exoribonucleases in eleven intensively studied bacterial species, using available transposon collections and genome-wide gene-deletion studies [33]–[50]. As described in detail in Materials and Methods, this summary represents our best effort to analyze publically available databases, taking into account possible misclassification of the essential nature of individual RNases (e.g., transposon mutagenesis can miss mutants that grow very slowly or tolerate certain insertions; gene inactivation cannot detect essential functions covered by multiple genes). Bacterial species were sorted according to phylogeny based on the amino acid sequence of each YbeY homolog; RNases were sorted according to their distribution, the most ubiquitous RNases being listed first.

This comparative analysis of RNases (Figure 1) reveals numerous patterns: a) YbeY is more often essential than not, indicating that the model organism *E. coli* is not always the most representative; b) the RNases of the postulated minimal bacterial genome [15] overlap well with the most ubiquitous RNases of both Gram-negative and Gram-positive bacteria, e.g. YbeY, RNase P, RNase III and PNPase; c) the majority of essential RNases are endonucleases, whereas bacterial exonucleases are functionally more redundant than endonucleases and are hence rarely essential; and d) the genome size of each organism correlates well with the number of major RNases, although the total number of essential RNases remains relatively constant with an average of five to six RNases. *E. coli* and *S. typhi* are extremes and show a remarkable redundancy of RNase genes with the highest number of total RNase genes and the lowest number of essential RNase genes, suggesting that many RNases share overlapping functions.

Overall, our comparative analysis of the essential nature of RNases in eleven different bacteria from a wide phylogenetic range allowed us to put YbeY firmly within a minimal core set of RNases, which overlaps with the set of RNases found in the minimal bacterial genome. Thus, it is not surprising that YbeY is essential in most pathogens that were included in our analysis. We chose to study YbeY in the model pathogen *V. cholerae* strain C6706, which is a clinical isolate of the seventh pandemic belonging to the O1 El Tor biotype and highly similar to the sequenced strain N16961 [51]. Of particular interest, its *ybeY* homolog is proposed to be essential [36].

### YbeY is essential for growth of *V. cholerae*

**Figure 2:**
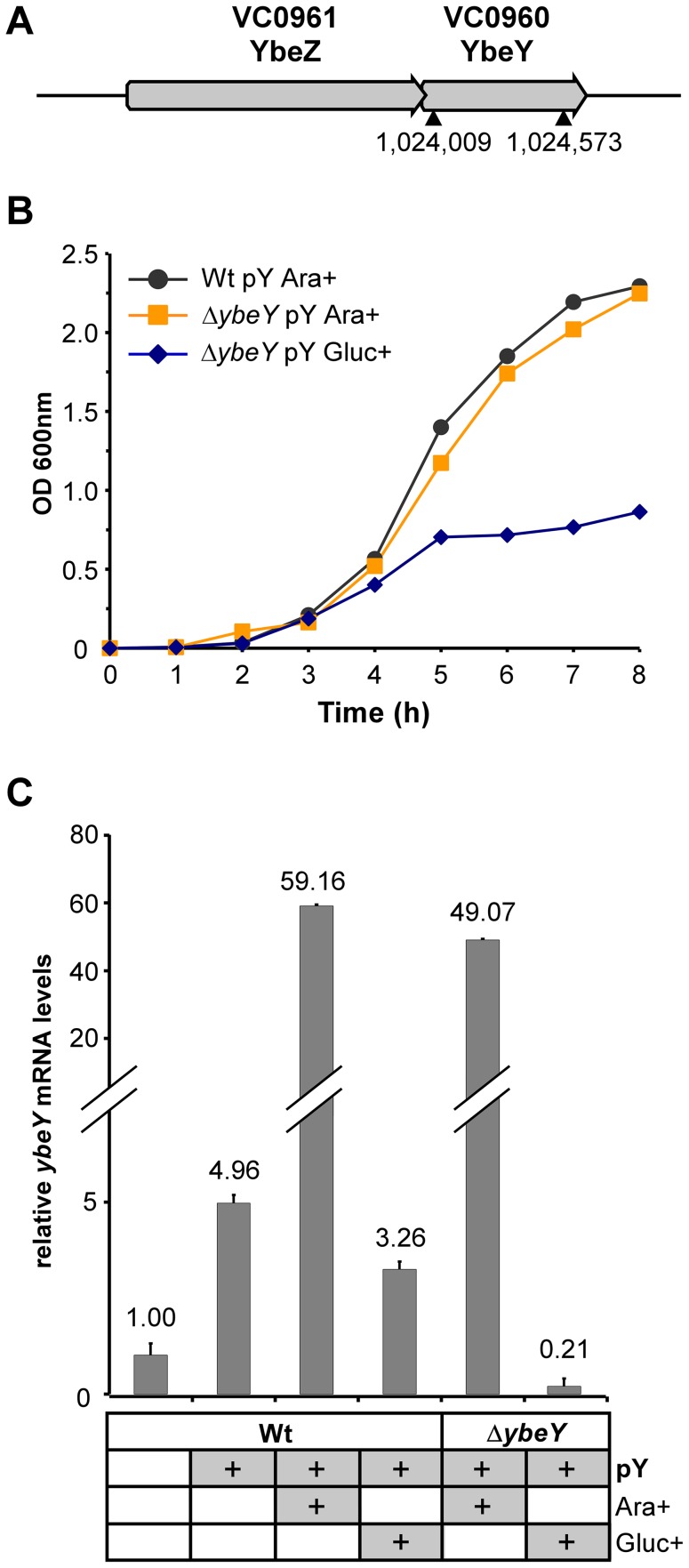
Generation of a Δ*ybeY* strain in *V. cholerae*. **A**) Genomic organization of the *ybeZ* (VC0961)-*ybeY* (VC0960) locus in the *V. cholerae* strain C6706. An in-frame deletion of *ybeY* was carried out in C6706; the position of the chromosomal *ybeY* deletion is indicated (▴). **B**) Growth analysis of the C6706 wild-type strain (Wt pY) and Δ*ybeY* pY mutant strain (Δ*ybeY* pY) transformed with a plasmid containing *ybeY* under control of an arabinose-inducible promoter. Cells were first grown in LB medium supplemented with arabinose and then subcultured in medium containing arabinose or glucose to obtain full induction or maximum depletion of YbeY, respectively. “pY” indicates that *ybeY* is expressed from a plasmid. Ara+, cells were grown in LB in the presence of arabinose. Gluc+, cells were grown in LB in the presence of glucose. **C**) RT-qPCR analysis of *ybeY* mRNA in the C6706 wild-type strain (Wt) and the Δ*ybeY* pY strain. Samples were taken from log phase cultures (OD_600_ 0.5), as shown above. The levels of *ybeY* mRNA were normalized to 5 S rRNA. The Wt strain grown in the absence of any carbon source and without the maintenance plasmid was set to 1.

A large-scale, saturating transposon library of *V. cholerae* showed no hits for the *ybeY* homolog, designated VC0960 [36]. To experimentally verify the essentiality of *ybeY* in *V. cholerae*, we attempted to create a non-polar deletion of *ybeY*, which is part of the *ybeZY* operon (Figure 2A), in *V. cholerae* C6706 using standard allelic exchange [52]. However, chromosomal *ybeY* could be deleted only in a strain carrying a copy of *ybeY* on the arabinose inducible vector pBAD18 (henceforth referred to as Δ*ybeY* pY) in the presence of 0.1% arabinose. In the absence of arabinose, no such non-polar deletion of *ybeY* could be obtained.

The growth rate of the Δ*ybeY* pY strain is identical to its parental strain in LB medium in the presence of 0.1% arabinose (denoted as Ara+) (Figure 2B). When the Δ*ybeY* pY strain was transferred to arabinose-free medium (denoted as Ara-) (Figure S1), cells showed a reduction in growth rate indicating YbeY depletion. If these cells were subcultured a second time into medium lacking arabinose (denoted as Ara-/Ara-) (Figure S1), cell growth was diminished to such an extent that we could not obtain enough cells for experimental measurements. An experimentally more convenient protocol for decreasing the expression of YbeY from pBAD18 is to shift the Ara+ cells to medium lacking arabinose but containing glucose, which decreases the 3′,5′-cyclic AMP levels, lowering expression from the catabolite-repressed P_BAD_ promoter. Thus, maximal depletion of YbeY was obtained by subculturing overnight cultures of the Δ*ybeY* pY strain grown in the presence of arabinose into medium that lacked arabinose but was supplemented with 0.2% glucose (denoted as Gluc+) (Figure 2B). After being switched to medium with glucose, Δ*ybeY* pY cells initially grow similar to the wild-type (Wt) but grow slower after mid-exponential phase and stop growing at a significantly lower optical density (OD_600_) in stationary phase. No cell death or loss of viability was observed in late-stationary phase after YbeY depletion when cells were plated on arabinose-containing solid media to determine colony-forming units (data not shown). Since simply inoculating overnight cultures of cells grown with arabinose into medium containing glucose (Gluc+) gave robust YbeY depletion, we have chosen these conditions for most experiments described in this study, as they allowed us to maximally deplete YbeY levels in a controlled fashion (see also Figure 2C) without affecting cell viability.

The levels of *ybeY* mRNA in *V. cholerae* wild-type and the plasmid-containing mutant strain were determined by RT-qPCR (Figure 2C). Notably, the presence of the pBAD18 derivative containing the extrachromosomal copy of *ybeY* (pY) led to a moderate increase in *ybeY* mRNA levels due to leaky transcription in the absence of arabinose or glucose. Expression of *ybeY* is strongly induced by arabinose (Ara+). In contrast, *ybeY* expression was reduced to 20% of the endogenous wild-type *ybeY* levels when arabinose was removed from the media and glucose was added (Gluc+).

Our ability to generate a *ybeY* deletion in *V. cholerae* C6706 only in the presence of a complementing plasmid and the striking decrease in growth rate upon YbeY depletion strongly support the conclusion that YbeY is essential for growth of *V. cholerae* in rich media.

### Depletion of YbeY in *V. cholerae* affects 16 S rRNA maturation and 70 S ribosome assembly

**Figure 3:**
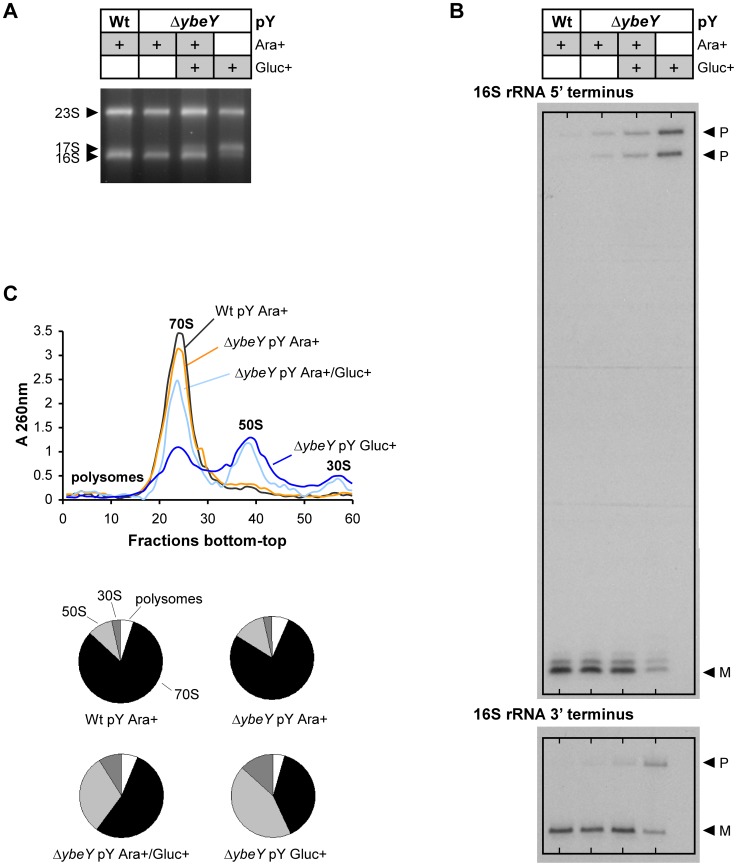
Analysis of rRNA and ribosome profiles in *V. cholerae* Δ*ybeY*. **A**) Total RNA was isolated from C6706 Wt pY and the Δ*ybeY* pY strain grown in the presence of arabinose or glucose as specified and analyzed by agarose gel electrophoresis. The positions of 23 S, 17 S, and 16 S rRNAs are indicated based on their mobility. **B**) The 5′ and 3′ termini of 16 S rRNA from C6706 Wt pY and the Δ*ybeY* pY strain were mapped as described in detail in Materials and Methods. “P” and “M” specify the positions of bands derived from the precursor and mature forms of 16 S rRNA. **C**) Ribosome profiles for C6706 Wt pY and the Δ*ybeY* pY strain (top); quantitation of polysomes, 70 S, 50 S and 30 S ribosomes (bottom pie charts). “pY” indicates that *ybeY* is expressed from a plasmid. Ara+, cells were grown in LB in the presence of arabinose. Gluc+, cells were grown in LB in the presence of glucose. Ara+/Gluc+, intermediate YbeY depletion by switching the carbon source of the Δ*ybeY* pY strain in early exponential phase from arabinose to glucose (for details see Materials and Methods).

Functional 70 S ribosomes are composed of 16 S, 23 S, and 5 S rRNAs that are processed from a larger RNA precursor. In *E. coli*, YbeY has been shown to be primarily involved in the maturation of both termini of 16 S rRNA and to also play a minor role in the 5′ maturation of 23 S and 5 S rRNAs [16]. Moreover, YbeY is essential for 16 S rRNA maturation at 45°C [18]. Very little is known about rRNA maturation in *V. cholerae* to date. To determine the role of *V. cholerae* YbeY in rRNA maturation, we isolated total RNA from the wild-type and the Δ*ybeY* pY strain (Figure 3A). Δ*ybeY* pY cells grown in the presence of arabinose (Ara+) exhibited a wild-type like rRNA profile. However, maximum depletion of YbeY in cells grown in the presence of glucose (Gluc+) showed strong accumulation of 17 S rRNA. We were able to detect an intermediate decrease of 16 S rRNA and a concomitant intermediate increase of 17 S precursor levels compared to the parental strain by switching the carbon source of the Δ*ybeY* pY strain in early exponential phase from arabinose to glucose (intermediate YbeY depletion; denoted as Ara+/Gluc+ in Figure 3A). Mapping of the 5′ and 3′ termini of all rRNAs confirmed the strong defect of 16 S rRNA maturation upon YbeY depletion, however no 23 S and 5 S rRNA processing deficiencies were detected (Figure 3B, S2). The latter shows that although *E. coli* and *V. cholerae* are considered to be fairly closely related species, the substrate specificity of individual RNases may be different as the distribution of RNase genes and their essentiality also differ (Table S1). Mapping of the 16 S rRNA 5′ end showed, as expected, two precursors since 4 of the 8 16 S rRNA genes have a ∼20-nucleotide deletion within the 5′ terminal precursor sequence in *V. cholerae* C6706.

**Figure 4:**
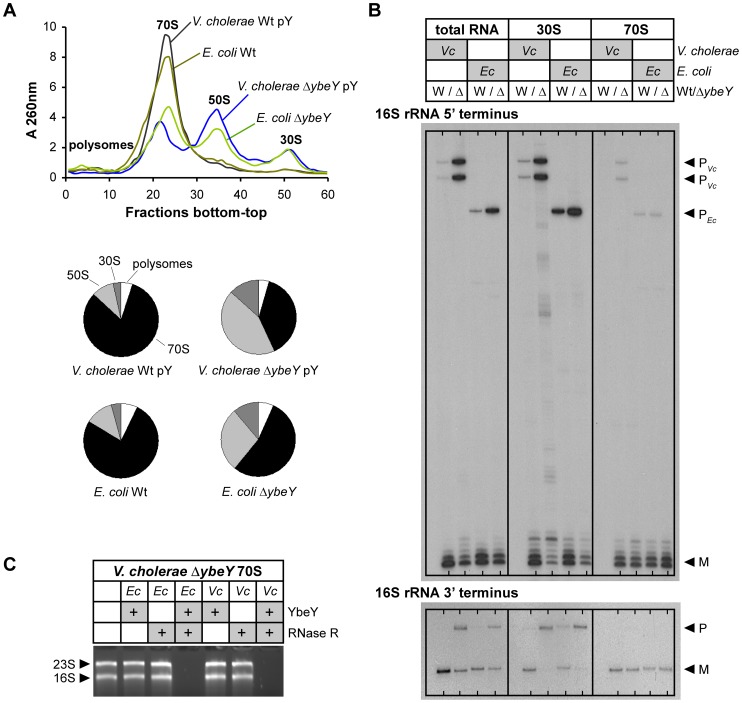
Comparison of ribosome profiles and 16*V. cholerae* Δ*ybeY* and *E. coli* Δ*ybeY*. **A**) Ribosome profiles for *V. cholerae* C6706 Wt pY and the Δ*ybeY* pY strain, and *E. coli* MC4100 Wt and the Δ*ybeY* strain (top); quantitation of polysomes, 70 S, 50 S and 30 S ribosomes (bottom pie charts). “pY” indicates that *ybeY* is expressed from a plasmid. *V. cholerae* Wt pY was grown in LB in the presence of arabinose (Ara+) and *V. cholerae* Δ*ybeY* pY was grown in LB in the presence of glucose for maximum depletion (Gluc+). *E. coli* cells were grown in LB medium without additional carbon source. **B**) Mapping of 5′ and 3′ termini of 16 S rRNA in total RNA, 30 S ribosomal subunits and 70 S ribosomes from C6706 Wt pY (W) and the Δ*ybeY* pY strain (Δ), and *E. coli* MC4100 Wt (W) and the Δ*ybeY* strain (Δ). rRNA was isolated from samples shown in **A**). “P” and “M” specify the positions of bands derived from the precursor and mature forms of 16 S rRNA. **C**) *In vitro* ribosome quality control by *V. cholerae* (*Vc*) and *E. coli* (*Ec*) YbeY together with RNase R. 70 S ribosomes of a *V. cholerae* Δ*ybeY* pY strain upon maximum YbeY depletion were incubated with YbeY, RNase R, or a mixture of YbeY and RNase R from *E. coli* or *V. cholerae* as indicated. The positions of 23 S and 16 S rRNAs are indicated based on their mobility.

The 16 S rRNA maturation defect also affects the ribosome pool as indicated by sucrose gradient analysis of ribosomes from *V. cholerae* wild-type and the Δ*ybeY* pY strain. Upon gradual depletion of YbeY, the level of intact 70 S ribosomes decreased accordingly and the level of individual 50 S and 30 S subunits increased compared to the parental strain (Figure 3C). Compared to an *E. coli* Δ*ybeY* strain in which the *ybeY* gene was completely deleted, we observed a more pronounced accumulation of individual ribosomal subunits in the *V. cholerae* Δ*ybeY* pY strain (Figure 4A), when *ybeY* mRNA levels are reduced to 20% of wild-type levels (Figure 2C, maximum YbeY depletion).

Mapping of both ends of 16 S rRNA in total RNA, 70 S ribosomes and 30 S subunits isolated from the *V. cholerae* Δ*ybeY* pY strain upon maximum YbeY depletion and from the *E. coli* Δ*ybeY* strain demonstrated that 30 S subunits contain mostly immature 16 S rRNA, whereas rRNA isolated from assembled 70 S ribosomes is mostly mature (Figure 4B). Total RNA samples, representing a mixture of free rRNA and rRNA from 30 S and 70 S ribosomes, also show strong maturation defects. These effects are similar in *V. cholerae* and *E. coli*. Observations made here are consistent with the assembly of immature or only partially matured 16 S rRNA into 30 S subunits. However, if such subunits are not processed further, as is the case in cells with reduced or no YbeY activity, they cannot be assembled efficiently into 70 S ribosomes. As a consequence, although the 70 S ribosome peaks in both the *V. cholerae* YbeY-depleted strain and the *E. coli* Δ*ybeY* strain (Figures 3C and 4A) are drastically reduced, they contain mostly mature 16 S rRNA (Figure 4B). Notably, the level of 17 S precursor in 70 S ribosomes isolated from *E. coli* Δ*ybeY* shown in this work (Figure 4B, 4C) is lower compared to our previous work [16], [18], though 16 S rRNA with mature termini is consistently the dominant species in 70 S ribosomes in contrast to 30 S ribosomal subunits. In the course of our work with Δ*ybeY* strains, we have observed that the absolute amount of precursor species can vary, depending on slight differences in culturing conditions, such as the level of aeration, source of LB medium etc. (data not shown).

YbeY of *E. coli* can degrade rRNA, mRNA and oligoribonucleotides *in vitro* [18]. Its active site has a highly conserved metal-coordinating histidine H3XH5XH motif that is characteristic to the UPF0054 protein family. The RNase activity of *V. cholerae* YbeY was confirmed by our demonstration that it was capable of degrading total *V. cholera* RNA, while its metal-dependency was confirmed by showing that the addition of EDTA inhibited this degradation (Figure S3A). Using an RNA hairpin and single-strand oligoribonucleotide substrates, we further demonstrated that *V. cholerae* YbeY is an endoribonuclease, whose substrate specificity is very similar to *E. coli* YbeY (Figure S3B, S3C). To improve *in vitro* activity and to prevent aggregation of purified *V. cholerae* YbeY, we used a plasmid that encodes both GroES and GroEL to increase chaperone activity. The RNase activity of purified *V. cholerae* YbeY was still slightly lower than that of purified *E. coli* YbeY, which could be due to the fact that YbeY was purified from *E. coli* BL21, a non-native host strain. In addition, we showed that YbeY together with RNase R of both *V. cholerae* and *E. coli* can degrade 70 S ribosomes isolated from the *V. cholerae* Δ*ybeY* pY strain grown under maximum YbeY depletion, but does not degrade *V. cholerae* wild-type ribosomes (Figure 4C, S3D). This is consistent with analogous experiments in *E. coli* [18], thereby demonstrating the conservation of the role of *V. cholerae* YbeY in ribosome quality control and the cross-species conservation of the YbeY-dependent ribosome quality control mechanism as *E. coli* enzymes recognize and degrade defective *V. cholerae* ribosomes.

### 16 S rRNA maturation in *V. cholerae* mutant strains carrying transposon insertions in genes encoding RNase E, RNase G, or RNase R

**Figure 5:**
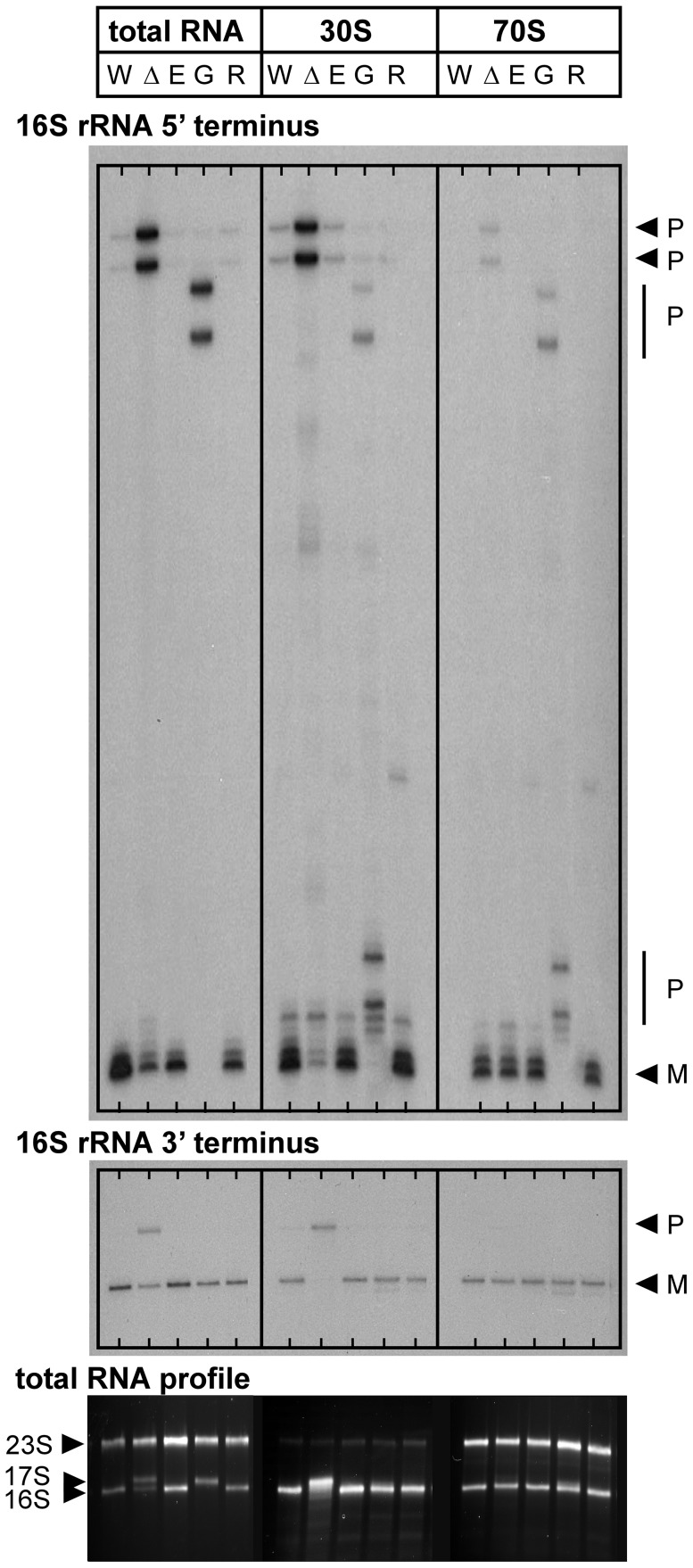
16*V. cholerae* mutant strains with transposon insertions in genes encoding RNase E (*rne-*CTD::Tn), RNase G (*rng*::Tn), or RNase R (*rnr*::Tn). Mapping of 5′ and 3′ termini of 16 S rRNA in total RNA, 30 S ribosomal subunits and 70 S ribosomes from C6706 Wt pY (W), Δ*ybeY* pY (Δ), *rne*-CTD::Tn (E), *rng*::Tn (G) and *rnr*::Tn (R); “P” and “M” specify the positions of bands derived from the precursor and mature forms of 16 S rRNA (top and middle panel). Analysis of total RNA by agarose gel electrophoresis; the positions of 23 S, 17 S and 16 S rRNAs are indicated based on their mobility (bottom panel). “pY” indicates that *ybeY* is expressed from a plasmid. *V. cholerae* Wt pY was grown in LB in the presence of arabinose (Ara+) and *V. cholerae* Δ*ybeY* pY was grown in LB in the presence of glucose for maximum depletion (Gluc+); all other strains were grown in LB medium without additional carbon source.

To analyze the involvement of *V. cholerae* YbeY in maturation of 16 S rRNA in comparison to other RNases that are functionally associated with 16 S rRNA maturation in *E. coli*, we compared the 5′ and 3′ termini of 16 S rRNA from *V. cholerae* wild-type, the *rne*-CTD::Tn [36], *rng*::Tn [36] and *rnr*::Tn mutant strains [36], and the Δ*ybeY* pY strain depleted of YbeY (Gluc+) (Figure 5). In *E. coli*, RNase E (*rne*) and RNase G (*rng*) participate in the two-step, sequential maturation of the 5′ end of 16 S rRNA [29]. Although RNase E is essential for growth in *E. coli*, several *rne*::Tn mutants were found in *V. cholerae* transposon libraries [36], [53]. However, all transposons were inserted downstream of the N-terminal domain (NTD) of RNase E that specifies the endonuclease activity, confirming the essentiality of its nuclease activity in *V. cholerae*. The C-terminal domain (CTD) functions as a scaffold for various interacting proteins that make up the degradosome and was recently shown to be required for binding to 30 S subunits of *E. coli* [31], [54]. Since multiple mutants with transposon insertions within the CTD of RNase E are available in *V. cholerae* [36], [53], a fully functional degradosome may not be essential for the viability of *V. cholerae*. The transposon of the representative *rne*::Tn mutant we chose to investigate further is located between segment A and the first RNA binding domain of RNase E [31]. Therefore, this mutant, designated as *rne*-CTD::Tn, lacks the full C-terminal scaffolding domain as confirmed by RT-qPCR (data not shown). In addition, a *rnr*::Tn mutant was examined as RNase R was shown in *E. coli* to participate, together with YbeY, in 70 S ribosome quality control and maturation of the 3′ end of 16 S rRNA [16], [18], [30].

The efficiency of 16 S rRNA 5′ end processing is reduced considerably upon depletion of YbeY as well as in the absence of RNase G, but is unaffected in both *rne*-CTD::Tn and *rnr*::Tn mutants (Figure 5, top panel). Notably, no mature 5′ end was detected in total RNA samples from the *V. cholerae rng*::Tn mutant, in contrast to *E. coli*, which still contains a substantial amount of the mature form in the absence of RNase G [29]. These results were confirmed by agarose gel electrophoresis, showing a 17 S-like precursor in total RNA of the *rng*::Tn mutant (Figure 5, bottom panel). Maturation of the 3′ end of 16 S rRNA is defective upon maximum depletion of YbeY in *V. cholerae* but not in the *rne*-CTD::Tn, *rng*::Tn and rnr::Tn mutants, demonstrating that YbeY is a key RNase required for processing of the critical 16 S rRNA 3′ end (Figure 5, middle panel).

In both the 30 S subunits and 70 S ribosomes of the *V. cholerae rng*::Tn mutant, additional, although still incomplete, processing of the 5′ end was observed, showing that low-level maturation occurs in the ribosomal context. The 70 S ribosome of the *V. cholerae rng*::Tn mutant contains immature 5′ ends, which is different from the *V. cholerae* YbeY-depleted Δ*ybeY* pY strain. This is consistent with the fact that the *V. cholerae rng*::Tn has no apparent growth phenotype (data not shown) and ribosomes with immature 16 S rRNA 5′ termini can function in contrast to ribosomes with immature 3′ termini [55]–[57].

Sucrose gradient analysis of *V. cholerae* ribosomes shows no defects in the absence of RNase G, RNase E:ΔCTD, and RNase R compared to the wild-type (Figure S4), possibly due to correct 16 S rRNA 3′ maturation. Therefore, among the RNases whose function we assessed, YbeY is the only RNase crucial for 3′ processing of 16 S rRNA and its assembly into 70 S ribosomes in *V. cholerae*.

### YbeY affects regulation of virulence associated small RNAs

Expression of critical virulence genes in various pathogens is often regulated by small RNAs (sRNAs). In many cases, regulation by sRNA depends on the RNA chaperone Hfq with a subsequent involvement of RNase E [58]. In *S. meliloti*, YbeY function clearly affected the regulation of certain sRNAs and their target mRNAs [21]. To investigate a possible role of *V. cholerae* YbeY in sRNA regulation, we selected five sRNAs thought to be important for pathogenicity: MicX and VrrA, which regulate the expression of outer membrane proteins [59], [60]; TarB, which targets the colonization factor TcpF [61] and the *Vibrio* seventh pandemic regulator VspR [62]; Qrr1–4 sRNAs, which control quorum sensing and those virulence genes that are regulated by quorum sensing [63]; and the regulator 6 S RNA, which binds to and thereby affects transcription of RNA polymerase σ^70^-driven genes [12].

**Figure 6:**
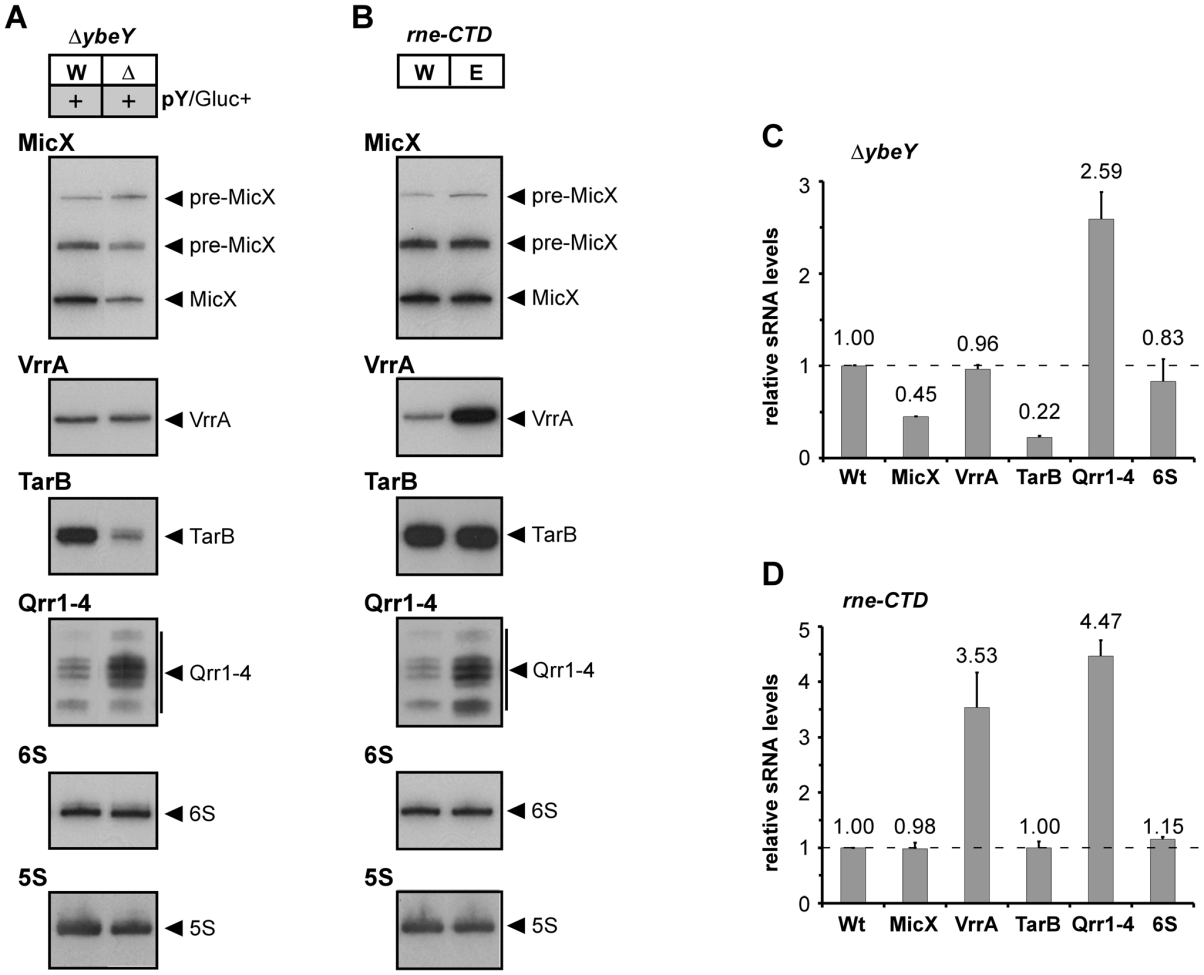
sRNA analysis in *V. cholerae* Δ*ybeY* and *rne*-CTD::Tn. Northern blot analysis of MicX, VrrA, TarB, Qrr1–4 and 6 S RNA in **A**) *V. cholerae* Δ*ybeY* pY and **B**) *V. cholerae rne*-CTD::Tn. Total RNA was isolated from C6706 Wt, Wt pY, Δ*ybeY* pY and *rne-*CTD::Tn grown in LB medium and analyzed by denaturing 6% polyacrylamide gel electrophoresis followed by Northern blot hybridization. The sizes of sRNAs were in accordance with published data. A 5 S rRNA-specific probe was used as a loading control. Hybridization signals were evaluated by phosphorimager analysis in **C**) and **D**); the levels of various sRNAs were normalized to 5 S rRNA. The Wt strain was set to 1. “pY” indicates that *ybeY* is expressed from a plasmid. Gluc+, cells were grown in the presence of glucose.

Expression patterns of MicX, VrrA, TarB, Qrr1–4 and 6 S RNA were monitored by Northern blot analysis of total RNA isolated from Δ*ybeY* pY cells upon maximum YbeY depletion and from *rne*-CTD::Tn cells (Figure 6). Steady state levels of MicX and TarB sRNAs were significantly reduced upon YbeY depletion compared to the parental strain, while levels of Qrr1–4 sRNAs were elevated (Figure 6A). In contrast, the *rne*-CTD::Tn mutant, which harbors an RNase E mutant lacking the docking domain for Hfq and other components of the degradosome [31], showed no changes in MicX and TarB sRNA levels but strongly increased VrrA and Qrr1-4 sRNA levels (Figure 6B). 6 S RNA levels did not change in either of the mutant strains (Figure 6A and B). Quantitation of sRNA levels was carried out using 5 S rRNA as a loading control (Figure 6C and D). Taken together, these results show a clear role of *V. cholerae* YbeY in sRNA regulation, different from that of the traditional Hfq/RNase E-mediated system.

### YbeY plays an important role in *V. cholerae* pathogenesis

**Figure 7:**
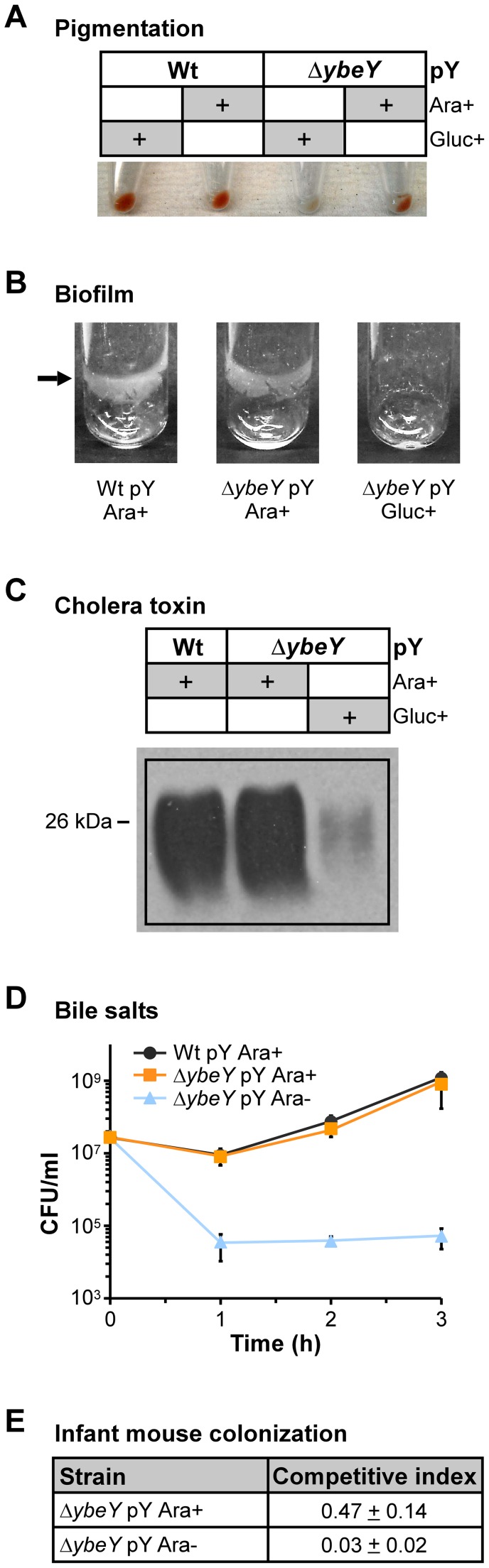
Reduced virulence in *V. cholerae* Δ*ybeY*. **A**) Depletion of YbeY leads to a deficiency of pigment formation. C6706 Wt pY and Δ*ybeY* pY cells were grown in LB medium in the presence of arabinose or glucose as indicated. When cells reached mid to late-log phase, a similar number of cells was spun down and the cell pellets were photographed. **B**) Depletion of YbeY results in a drastic decrease of biofilm formation in C6706 Δ*ybeY* pY cells. Cultures were grown statically in LB medium supplemented with arabinose or glucose as indicated. **C**) Depletion of YbeY leads to reduced levels of cholera toxin (CT). C6706 Wt pY and Δ*ybeY* pY cells were grown in LB medium in the presence of arabinose or glucose. The amount of CT was estimated in the supernatants of cultures by western blot analysis using a CT-specific antibody. Equivalent amounts of protein were applied per lane. **D**) Depletion of YbeY leads to sensitivity towards bile salts. C6706 Wt and Δ*ybeY* pY cells were grown in LB medium supplemented with 2.0% of bile salts and arabinose as specified. Samples were taken at the indicated times, and the number of colony forming units (CFU) was determined. **E**) Depletion of YbeY leads to reduced colonization of intestines in infant mice. Competitive indexes show the ability of C6706 Δ*ybeY* pY cells to colonize the infant mouse intestine relative to C6706 Wt and equal 1 upon full complementation. C6706 Δ*ybeY* pY cells were grown in the presence or absence of arabinose as indicated.

Certain RNases are important for pathogens to survive the fluctuating conditions during host infection and to control the expression of many virulence factors [64]. We have shown YbeY to be an RNase in *V. cholerae* that plays a role in regulating the levels of several virulence-associated sRNAs. To study YbeY's role in *V. cholerae* pathogenesis, we investigated the effect of YbeY's depletion on a number of specific virulence factors of *V. cholera*e. Initially, we observed an apparent loss of pigmentation in the *ΔybeY* pY strain upon maximum YbeY depletion to 20% of wild-type YbeY levels (Figure 7A). Also, overnight biofilm formation in static LB cultures was dramatically reduced upon maximum YbeY depletion, compared to wild-type and to *ΔybeY* pY cultures grown in the presence of arabinose (Figure 7B). Additionally, depletion of YbeY significantly reduced CT production of Δ*ybeY* pY cells (Figure 7C). Exposure to bile salts resulted in a two to four order of magnitude drop in survival of the *ΔybeY* pY strain upon YbeY depletion, 1 to 3 hours after cells were shifted to media lacking arabinose (Figure 7D). Finally, as depletion of YbeY clearly affects multiple virulence factors, we assessed YbeY's overall ability to colonize and persist in an infant-mouse colonization model (Figure 7E). Using a competition assay, we grew the wild-type and *ΔybeY* pY strain in the presence or absence of arabinose prior to inoculation of infant-mice. As shown in Figure S1, depletion of YbeY without glucose results in an intermediate growth phenotype allowing us to obtain an equal number of cells of the wild-type and *ΔybeY* pY mutant strain in early exponential phase. We found a striking reduction in colonization by the Δ*ybeY* pY strain relative to the parental strain, when both strains were grown in the absence of arabinose prior to inoculation (Figure 7E). Only a minor colonization defect was observed with strains grown in the presence of arabinose prior to inoculation. This lack of complete complementation is probably due to the absence of arabinose in the intestinal tract of mice, allowing for some/slight YbeY depletion. Taken together, these results suggest that YbeY plays a critical role during multiple stages of the *V. cholerae*'s infection cycle.

### YbeY plays a central role in stress regulation of *V. cholerae*

**Figure 8:**
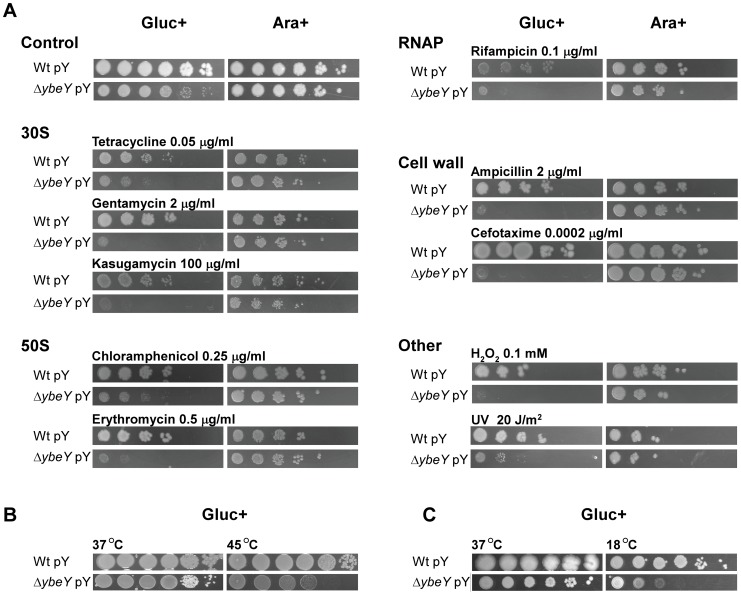
Sensitivity of *V. cholerae* Δ*ybeY* to various stresses. **A**) C6706 Wt pY and Δ*ybeY* pY cells were grown overnight in LB medium supplemented with arabinose, subsequently diluted to an OD_600_ 0.1 and then spotted as a dilution series (1∶10) onto LB plates supplemented with various antibiotics or H_2_O_2_ or irradiated with UV as indicated. Cells were grown in the presence of glucose (Gluc+) or arabinose (Ara+) as indicated. **B**) To determine heat-sensitivity of C6706 Wt pY and Δ*ybeY* pY cells, early exponential cultures supplemented with glucose were incubated for 3 h at 37°C and 45°C before spotting as a dilution series (1∶10) onto LB plates supplemented with glucose (Gluc+). Cells were then grown for 18 h at 37°C. **C**) To determine cold-sensitivity of C6706 Wt pY and Δ*ybeY* pY cells, cultures were spotted as a dilution series (1∶10) onto LB plates supplemented with glucose (Gluc+). Cells were then grown for 2 days at 37°C and 18°C as indicated.

The phenotype of *ΔybeY* deletion mutants in *S. meliloti* and *E. coli* is extremely pleiotropic, as they exhibit a strong sensitivity to a wide range of physiological stresses [14], [16]. To analyze the importance of YbeY function in protecting *V. cholerae* against stress, we tested the sensitivity of the *V. cholerae ΔybeY* pY strain to a broad variety of stresses targeting key cellular processes after YbeY had been depleted by growth in glucose. These stresses include antibiotics targeting protein synthesis, transcription, and cell wall synthesis, the oxidative stress H_2_O_2_, the DNA-damaging agent ultraviolet radiation and heat (Figure 8).

Cell growth was tested on plates by serial dilution of overnight cultures of *V. cholerae* wild-type and *ΔybeY* pY cells grown in the presence of arabinose; to deplete YbeY, glucose was present in the plating medium as indicated. Under non-stress conditions (37°C), *ΔybeY* pY showed similar growth upon YbeY depletion compared to wild-type, although mutant colonies at the highest dilutions were smaller than wild-type colonies due to their reduced growth rate (Figure 8A). Although *ybeY* is essential, no cell death was observed under these conditions, most likely because *ybeY*'s leaky expression still reaches approximately 20% of the endogenous wild-type *ybeY* levels (Figure 2B).

In general, cell growth of the *V. cholerae ΔybeY* pY strain on plates supplemented with glucose and one of several stress agents was drastically reduced compared to the parental strain (Figure 8A). Since YbeY has a major role in 16 S rRNA maturation, we tested three different antibiotics that target the 30 S ribosomal subunit each in a different way, tetracycline, gentamycin and kasugamycin. The *ΔybeY* pY strain showed a severe increase in sensitivity upon YbeY depletion to all of these antibiotics. A similar effect was observed with antibiotics targeting the 50 S ribosomal subunit, chloramphenicol and erythromycin. Additionally, we found that the *ΔybeY* pY strain also exhibited increased sensitivity to inhibition of RNA synthesis by rifampicin and cell wall synthesis by both ampicillin and cefotaxime. As *V. cholerae* is exposed to oxidative stress during host infection and UV radiation outside of the host, both stresses were tested as well, showing a consistent decrease in protection against oxidative and UV stress upon YbeY depletion (Figure 8A).

**Figure 9:**
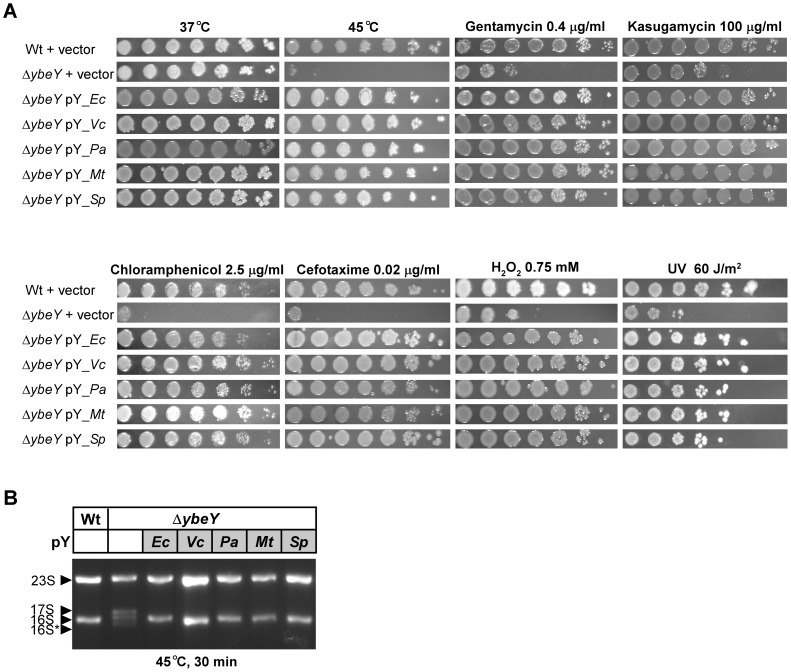
Complementation of *E. coli* Δ*ybeY* by *ybeY* of various pathogens. **A**) *E. coli* MC4100 Wt pY and the Δ*ybeY* pY strain grown in LB medium were serially (1∶10) diluted and spotted onto LB plates containing various antibiotics or H_2_O_2_, treated with heat (45°C) or UV (60 J/m^2^) as indicated. “pY_Ec” refers to a plasmid expressing *ybeY* of *E. coli*, similarly *V. cholerae* N16961 (pY_Vc), *P. aeruginosa* PAO1 (pY_Pa), *M. tuberculosis* H37Rv (pY_Mt), and *S. pneumoniae* R6 (pY_Sp). **B**) Total RNA was isolated from *E. coli* MC4100 transformed with the empty vector, and the *E. coli* Δ*ybeY* strain transformed with plasmids containing *ybeY* from various bacteria as indicated. RNA was analyzed by agarose gel electrophoresis. The positions of 23 S, 17 S, 16 S and 16 S* rRNAs are indicated based on their mobility.

In *E. coli*, YbeY is an important factor for rRNA maturation even at 37°C but becomes essential upon temperature shift to 45°C as shown by a complete loss of mature 16 S rRNA and cell viability [16], [18], [19]. YbeY depletion in a *V. cholerae ΔybeY* pY strain grown in glucose also causes a growth defect at 45°C after 3 hours compared to wild-type (Figure 8B), however only a two to three orders of magnitude drop in survival was consistently observed, in contrast to *E. coli*'s overall loss of viability at elevated temperatures (see Figure 9A). This difference might in part be *V. cholerae* specific but may also be due to the low background levels of YbeY still present in the *V. cholerae* depletion strain. Overexpression of *ybeY* does not provide additional heat protection to *V. cholerae* because the wild-type strain with and without the *ybeY* overexpression plasmid showed a similar level of heat tolerance (data not shown). Interestingly, a cold-sensitive phenotype was found upon depletion of YbeY in a *V. cholerae ΔybeY* pY strain at 18°C (Figure 8C). A similar cold-sensitivity was not observed in *E. coli*, further illustrating the disparity in response to changes in temperature between *V. cholerae* and *E. coli* depleted of YbeY.

### YbeY's activity in stress regulation and rRNA maturation is highly conserved among evolutionary distant pathogens

YbeY is a highly conserved protein found in almost all sequenced bacteria. To determine the level of functional conservation, we complemented the *E. coli ΔybeY* strain – grown under a variety of stress conditions – using constitutively expressed *ybeY* of four distantly related human pathogens, *V. cholerae*, *P. aeruginosa*, *M. tuberculosis* and *S. pneumoniae*. Of these representative YbeY orthologs, only the catalytic region, characterized by the H3XH5XH motive, shows a high level of conservation as illustrated by an alignment of all four YbeY proteins compared to *E. coli* YbeY (Figure S5). Compared to *E. coli* YbeY, the *V. cholerae* ortholog is 76% identical over the entire length of the protein, whereas the orthologs of *P. aeruginosa*, *M. tuberculosis*, and *S. pneumoniae* are 58%, 37%, and 33% identical, respectively.

The striking heat phenotype at 45°C of the *E. coli ΔybeY* strain could be fully complemented by all four pathogenic orthologs of YbeY (Figure 9A). The growth defect of the *E. coli ΔybeY* strain on plates containing antibiotics targeting the 30 S ribosomal unit, 50 S ribosomal unit, and cell wall synthesis could also be rescued by all four orthologs. Protection against oxidative and UV stress was restored as well. Consistent with the growth recovery at 45°C, the prominent defect of 16 S rRNA maturation at 45°C in the *E. coli ΔybeY* strain could also be rescued by all four pathogenic orthologs of YbeY (Figure 9B). Thus, the function of YbeY in stress regulation and rRNA maturation is well conserved among distantly related pathogens.

## Discussion

**Figure 10:**
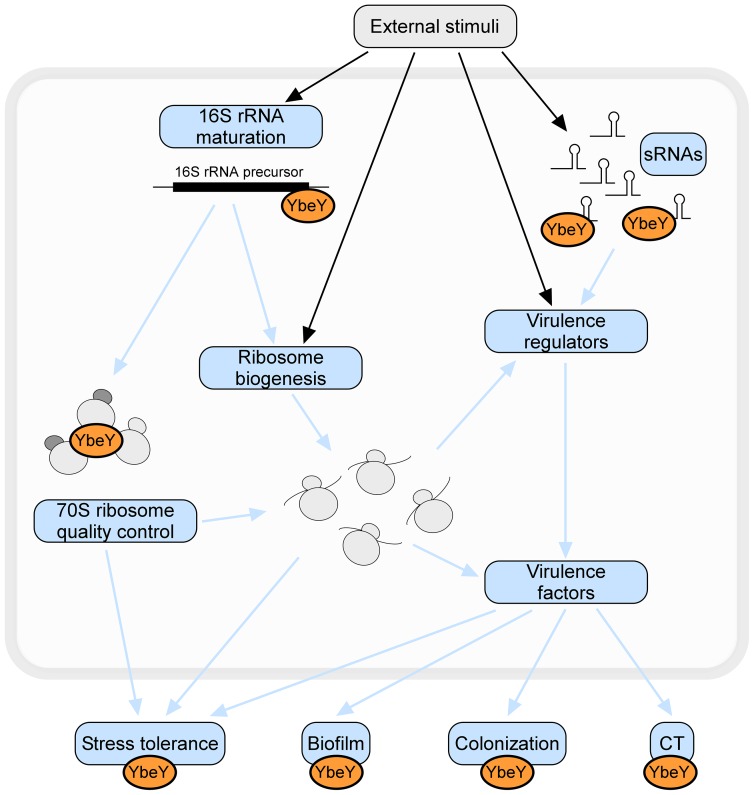
The many roles of YbeY. YbeY is a highly conserved essential RNase in *V. cholerae*, playing a key role in RNA processing, 70 S ribosome quality control, stress regulation and virulence. Using a *V. cholerae* mutant strain partially depleted for YbeY, we show that YbeY is crucial for 16 S rRNA 3′ end maturation and assembly of functional 70 S ribosomes. YbeY is also involved in the regulation of virulence-associated sRNAs and its depletion leads to reduced virulence, exemplified by reduced biofilm formation, decreased colonization of mouse intestines and decreased cholera toxin (CT) production. The regulatory role of YbeY in expression of various virulence genes (e.g. *Vibrio* exopolysaccharides, TcpA-F, AcfA-D, CtxA-B) could be direct or indirect via one the virulence regulators (e.g. ToxT, HapR). Depletion of YbeY also affects the overall cell viability and fitness and increases sensitivity towards a variety of stresses (e.g. temperature, UV, oxidative stress, antibiotics or bile salts). Considering YbeY's multifaceted role in several essential cellular processes and its conserved function across species, YbeY represents an attractive new target for antimicrobials.

We have shown that YbeY is an essential RNase in the pathogen *V. cholerae*. Depletion of YbeY: i) results in severe defects in 16 S rRNA maturation, ribosome assembly, and stress regulation, ii) affects regulation of virulence-associated sRNAs, and iii) reduces overall pathogenesis (Figure 10). Moreover, we found that key functions of YbeY's multifaceted activity are highly conserved among pathogens.

### YbeY is an essential RNase in *V. cholerae* playing a central role in RNA metabolism

YbeY is the first endoribonuclease of *E. coli* shown to play a direct or indirect role in processing of the 16 S rRNA 3′ terminus [16], [18]. However, most of what we know about rRNA processing comes primarily from studies in *E. coli* [31], [65], while relatively little information is available in other organisms. Here, we have shown that *V. cholerae* YbeY is also a metal-dependent endoribonuclease, although in contrast to *E. coli*, *ybeY* is essential in *V. cholerae* (Figure 2). Gradual depletion of YbeY in *V. cholerae* results in gradual accumulation of 16 S rRNA precursors that are defective in maturation of both ends, along with a gradual decrease in the efficiency of ribosome assembly (Figure 3). Similar to *E. coli*, where YbeY plays a minor role in 5′ maturation of 23 S and 5 S rRNAs [16], YbeY does not appear to be required for the maturation of 23 S and 5 S rRNAs in *V. cholerae*. We also examined 16 S rRNA processing in *V. cholerae* mutants of RNases associated with 16 S rRNA maturation in *E. coli*, the 5′ end processing RNases E and G and RNase R. The latter exoribonuclease plays an important role in 70 S ribosome quality control together with YbeY [18] and has been shown to largely bind to the ribosome close to the 5′ end of 16 S rRNA [66]. Using an *rne*:Tn mutant (*rne*-CTD::Tn) that contains the essential N-terminal nuclease domain but lacks the C-terminal degradosome scaffold [31], we have shown that an intact RNase E nuclease domain is sufficient for 5′ processing of 16 S rRNA (Figure 5). As expected, a 5′ processing defect was observed in the *rng*::Tn mutant. 3′ maturation was not affected in the *rne*-CTD::Tn and *rng*::Tn mutants, and lack of RNase R had no effect on 5′ or 3′ processing.

Similar to *E. coli* [16], depletion of YbeY leads to processing defects at both the 5′ and 3′ ends of 16 S rRNA (Figure 4). The role of YbeY in 5′ maturation of 16 S rRNA is likely indirect. It is possible that 5′ processing of 16 S rRNA is dependent on 3′ processing, which is in accord with a recent report showing that in *E. coli* 5′ processing proceeded much less efficiently in the absence of four exoribonucleases thought to be involved in 3′ maturation of 16 S rRNA [30]. Moreover, the 30 S particles in the YbeY depleted samples contained mostly 16 S rRNA precursors, indicating that correct 16 S rRNA maturation may be required for efficient 70 S ribosome assembly and that the final maturation of 16 S rRNA occurs on the ribosome.

We also demonstrated that 70 S ribosome quality control mediated by YbeY and RNase R is not limited to *E. coli* [18]. Defective *V. cholerae* 70 S ribosomes were degraded by the combined action of *V. cholerae* YbeY and RNase R (Figure 4 and S3). Similar to YbeY, RNase R is also present in most representative pathogens (Figure 1), suggesting the possibility of a universal ribosome quality control mechanism executed jointly by an endonuclease and an exonuclease. Since 70 S ribosomes isolated from a *V. cholerae* Δ*ybeY* strain containing 16 S rRNA with mostly mature termini are completely degraded by YbeY and RNase R, we can assume that this quality control mechanism is not solely triggered by unprocessed rRNA. In agreement with our previously proposed model [18], YbeY alongside RNase R may act as a sensor of rRNA perturbations caused by misfolding or lack of certain base modifications, rRNA damage by environmental factors, or an imbalance in ribosomal proteins or ribosome maturation factors in defective 70 S ribosomes. In addition, it is quite possible that some of the factors that trigger the YbeY-dependent ribosome quality control described above may also contribute to the defects in ribosome assembly observed in YbeY-depleted strains.

YbeY's function in RNA metabolism also extends to sRNA regulation as reported earlier in *S. meliloti* [21]. YbeY proteins share structural similarities with the MID domain of Argonaute proteins, illustrating a conceptual parallel between prokaryote and eukaryote sRNA pathways [21]. Regulatory RNAs are a powerful means of regulating gene expression at multiple levels, allowing bacteria to respond quickly to environmental changes. Often, sRNAs depend on the RNA chaperone Hfq and RNA degradation by RNases, such as RNase E or PNPase [5], [58], [67]. Hfq was shown to be important for stress tolerance and virulence in many pathogens [68]. Currently, around 20 sRNAs have been experimentally confirmed in *V. cholerae* [69], [70]. Here, we show for the first time, that YbeY is also involved in the regulation of several virulence-associated sRNAs in *V. cholerae*, namely MicX, TarB and Qrr1–4 (Figure 6A). It is currently not known, whether YbeY requires Hfq to do so. In *V. cholerae*, regulation of quorum sensing sRNAs and TarB is mediated by Hfq [62], [71]. One could easily envision that YbeY functions alongside Hfq and replaces the function of the commonly used RNase E for a certain subset of sRNAs. Conversely, YbeY may also target certain sRNAs in an Hfq-independent manner as it has been shown for PNPase in *E. coli* [72]. Moreover, consistent with current knowledge of sRNA regulation we observed differential sRNA levels of VrrA and Qrr1–4 in the *V. cholerae* RNase E mutant which lacks the degradosome scaffold domain (*rne*-CTD::Tn) (Figure 6B), but saw no changes for sRNAs MicX and TarB that are targeted by YbeY. Although the essential nuclease activity of RNase E is maintained in *rne*-CTD::Tn, the docking site for various degradosome components such as Hfq and PNPase is absent, impeding the RNase E-mediated recruitment of interacting partners that are required for certain sRNAs. These data hence establish YbeY as an important player in sRNA regulation alongside Hfq, RNase E and PNPase, and add to its key role in bacterial RNA metabolism.

### YbeY affects multiple virulence factors and the overall stress response of *V. cholerae*

Virulence factors are gene products that improve bacterial survival during host infection. We demonstrate that depletion of YbeY impedes *V. cholerae*'s virulence by affecting virulence factors at multiple stages along the pathogenesis cycle (Figure 7). First, YbeY depletion reduces the level of pigmentation. Pigments like melanin protect cells against UV radiation, which is a major source of stress for pathogens outside the host and was previously shown to regulate expression of virulence factors [73]. Second, biofilm formation is drastically reduced upon YbeY depletion. Biofilms are highly organized microbial communities that are characterized by the ability to resist diverse stresses like antibiotics and host immune systems [74]. Biofilms are central to pathogenesis of many bacterial infections [75]. Third, YbeY depletion significantly reduces cholera toxin (CT) production. Colonization of the small bowel by *V. cholerae* must occur before CT is secreted into the intestinal epithelial cells and causes severe diarrhea. Therefore, YbeY-depleted cells that colonize the intestine are substantially less toxic than the parental strain. Fourth, lack of YbeY highly sensitizes the *V. cholerae ybeY* mutant to bile salts, which are antimicrobial substances present in the small intestines of mammals and one of the first stress factors encountered by the bacteria after passage through the acidic stomach. Lastly, intestinal colonization by the *V. cholerae ybeY* mutant is almost completely lost upon YbeY depletion in competition with the wild-type strain in infant mice, illustrating the overall reduced *in vivo* fitness or reduced ability to establish an infection. This is consistent with YbeY being one of the 507 genes that are highly expressed in both stool samples from cholera patients and during mid-exponential growth in rabbit ileal loops compared to growth in LB [76], [77]. Virulence defects observed upon YbeY depletion in *V. cholerae* could be partly indirect, since YbeY affects ribosome biogenesis and thereby globally affects protein synthesis.

The reduced virulence of the *ybeY* mutant is, however, opposite to that expected from the relative fall in TarB levels and rise in Qrr1–4 levels upon YbeY depletion; low TarB and elevated Qrr1–4 levels generally correlate with increased expression of specific virulence genes [61], [62], [71], [78]. Virulence, as defined by successful penetration of the host's defense mechanisms, colonization and toxigenesis, is nonetheless an intricate phenomenon and represents the net result of the coordinated expression of hundreds of genes including sRNAs, that are subject to multiple regulatory circuits [10], [79], [80]. While YbeY's exact role in the regulation of virulence-associated sRNAs and their effect on various virulence factors is unclear, YbeY evidently plays a crucial role in *V. cholerae* pathogenesis.

Stress adaptation is key to establishing a successful infection. YbeY was originally discovered in *S. meliloti* as a stress regulator crucial to establish a chronic intracellular infection required for the nitrogen-fixing symbiosis within its host plant *Medicago sativa* [14]. In *E. coli*, deletion of *ybeY* causes a pleiotropic stress phenotype, especially to heat [16]. Hence, YbeY was initially identified as a heat shock protein [20]. In *V. cholerae*, we showed that YbeY depletion drastically impairs the stress response to a wide variety of stress agents. Its remarkably pleiotropic phenotype (Figure 8A) distinguishes it from most RNase mutants studied so far, which typically show fewer phenotypic traits. However, YbeY of *V. cholerae* does not act like a heat shock protein. Depletion of YbeY results only in a modest decrease of cell survival after heat treatment compared to the wild-type (Figure 8B). On the other hand, unlike in *E. coli*, a modest cold-sensitive phenotype was found upon YbeY depletion in *V. cholerae ybeY* at 18°C (Figure 8C). Cold-sensitivity is a characteristic of ribosome assembly defects, since lower temperatures can stabilize incorrect ribosome intermediates [81], and is a well-established phenotype of RNase mutants lacking PNPase, RNase PH or RNase R [82], [83]. Hence, cold-sensitivity is in agreement with YbeY's key role in RNA metabolism.

### YbeY is part of a core set of bacterial RNases representing an attractive antimicrobial drug target

Based on high sequence conservation, YbeY is found in virtually all sequenced bacteria. Orthologs of *ybeY* are much less conserved in eukaryotes. So far, no orthologs could be identified in sequenced Fungi and Archaea although they are present in some higher eukaryotes, including humans. We have demonstrated here that YbeY's function in stress regulation and rRNA maturation is highly conserved in bacteria as *ybeY* orthologs of four distantly related pathogens, *S. pneumoniae*, *M. tuberculosis*, *P. aeruginosa*, and *V. cholerae*, could fully complement an *E. coli ΔybeY* mutant subjected to a variety of stress agents and could correctly mature 16 S rRNA of *E. coli* during heat treatment (Figure 9). This is notable as conserved proteins do not always retain their specific characteristics, e.g. MazG of *M. tuberculosis*. This conserved NTP-pyrophosphohydrolase was shown to have a distinct function in *M. tuberculosis* compared to *E. coli* in response to starvation and oxidative stress [84].

Using a comprehensive phylogenetic analysis of major RNases in a selection of important bacterial pathogens (Figure 1), we showed that YbeY is more often essential than not and is part of a minimal core set of RNases, consistent with its remarkable level of functional conservation. While the overarching mechanisms by which organisms process RNA are conserved, our analysis also shows the disparity of RNases and their essentiality among different organisms. Thus, RNA maturation and degradation machineries necessitate the implementation of a set of RNases, which may vary from organism to organism, to fulfill all required functions. Altogether, the relatively low number of essential RNases underlines functional redundancy within groups of RNases and highlights the fundamental role of YbeY as a bacterial RNase.

Novel drug targets are crucial to combat the rapid rise of antibiotic resistance. The majority of today's antibiotics have been developed against essential components of central cellular pathways. New potential targets worth evaluating include bacteria-specific proteases, aminoacyl-tRNA synthetases, RNA-modification enzymes, and RNases. For example, the essential process of RNA turnover via RNases is considered rich in candidate antimicrobial targets [63]. The RNase YbeY represents an important new promising drug target. We have demonstrated that YbeY in *V. cholerae* plays an essential role in various aspects of RNA metabolism and overall pathogenesis (Figure 10). Depletion of YbeY in *V. cholerae* limits growth and increases the organism's sensitivity towards stress, making YbeY well suited for the development of target-specific antibiotics in whole-cell screening assays based on differential sensitivity of target-depleted and complemented strains. Such an approach was used successfully to discover platensimycin, a new class of broad-spectrum antibiotics for Gram-positive bacteria [85]. The availability of a suitable model host is also crucial for early antibiotic development [86]. Since depletion of YbeY in *V. cholerae* significantly reduces colonization of the pathogen in infant mice as well as expression of numerous virulence factors, a mouse model can be adapted for such a purpose. Although functionally associated with a well-established antibiotic target, the ribosome (Figure 10), YbeY is so far unexploited as a drug target and its use as a drug target might lead to the discovery of completely novel antibiotic scaffolds. A YbeY-specific inhibitor could also be used in combination with other antibiotics for enhanced antimicrobial activity, similar to combining silver and vancomycin [87] or rifampicin and acyldepsipeptide that activates the ClpP protease [88]. Considering YbeY's high level of conservation, its essential nature in many pathogens and its ability to sensitize pathogens by disrupting stress tolerance and virulence, a YbeY-specific antibiotic could have broad-spectrum antimicrobial activity.

## Materials and Methods

### Ethics statement

The animal experiments were performed with protocols approved by Harvard Medical School Office for Research Protection Standing Committee on Animals. The Harvard Medical School animal management program is accredited by the Association for the Assessment and Accreditation of Laboratory Animal Care, International (AAALAC), and meets National Institutes of Health standards as set forth in the Guide for the Care and Use of Laboratory Animals (DHHS Publication No. (NIH) 85–23 Revised 1996). The institution also accepts as mandatory the PHS Policy on Humane Care and Use of Laboratory Animals by Awardee Institutions and NIH Principles for the Utilization and Care of Vertebrate Animals Used in Testing, Research, and Training. There is on file with the Office of Laboratory Animal Welfare (OLAW) an approved Assurance of Compliance (A3431-01).

### General

DNA and RNA-DNA chimeric oligonucleotides were obtained from IDT or Eurofins MWG|Operon. Sequences of oligonucleotides used in this study are summarized in Table S1.

### Genome analysis of essential RNases in human pathogens

RNase genes were identified as putatively essential based on comparison of various deletion or transposon libraries in *V. cholerae* C6706 ([36], [53]; this work), *E. coli* MG1655 [39], [40], *S. typhi* Ty2 [37], [45], *H. influenzae* Rd KW20 [33], [34], *P. aeruginosa* PA14 ([46]; http://ausubellab.mgh.harvard.edu/cgi-bin/pa14/home.cgi), *B. subtilis* 168 [44], *S. aureus* NCTC 8325 [38], *S. pneumoniae* TIGR4 [48], *M. tuberculosis* H37v [42], [49], [50], *H. pylori* G27 [47], and *M. genitalium* G37 [41]. RNases were designated as essential if no deletion or transposon mutant was available for the respective open reading frame. For RNase E, several libraries contained mutants with transposon insertions in the second half of the gene corresponding to the C-terminal docking domain of RNase E, but no transposon insertions in the first half of the gene which corresponds to the essential RNase domain of RNase E [31]; in these cases, RNase E was termed essential. RNases were designated as non-essential when multiple deletion or transposon mutants were available, with transposon insertions randomly distributed within the respective open reading frame. The identity of individual RNases was confirmed by BLAST searches (NCBI).

### Strain construction and DNA manipulations

To generate a non-polar deletion of VC0960, we first constructed a *V. cholerae* C6706 strain carrying a copy of VC0960 on the arabinose inducible vector pBAD18 [89], named pY in the main text. The deletion of VC0960 (chromosomal location 1,024,009–1,024,573) was performed in the presence of arabinose as previously described [52].

The complementation plasmids expressing YbeY of various organisms were constructed by PCR amplification of each *ybeY* homolog from chromosomal DNA (i.e. b0659 of *E. coli* MG1655, VC0960 of *V. cholerae* C6706, PA3982 of *P. aeruginosa* PAO1, SPR0869 of *S. pneumoniae* R6, and Rv2367c of *M. tuberculosis* H37v), and subsequently cloned into pBR322 under control of the constitutive tetracycline promoter.

DNA manipulations were performed according to the methods of Sambrook [90] and cloning products were sequence-verified.

### Bacterial strains and growth conditions

The bacterial strains used for this work are *V. cholerae* wild-type C6706 [91], *V. cholerae* C6706 *ΔybeY* pY (this work), *V. cholerae* C6706 *rne*-CTD::Tn ([36], Mutant ID EC2413, Genomic locus 2.183.265), *V. cholerae* C6706 *rng*::Tn ([36], Mutant ID EC15315, Genomic locus 445.329), *V. cholerae* C6706 *rnr*::Tn ([36], Mutant ID EC9412, Genomic locus 2.768.302), *E. coli* wild-type MC4100 [92], and *E. coli* MC4100 *ΔybeY* [16]. *V. cholerae* and *E. coli* were cultured in LB medium (1% tryptone, 0.5% yeast extract, 0.5% NaCl) at 37°C when used for RNA isolation, ribosome isolation, protein purification or stress tests. *V. cholerae* strains were maintained in LB medium supplemented with 0.1% arabinose (Ara+; YbeY induction) or 0.2% glucose (Gluc+; YbeY depletion) as indicated. For depletion of YbeY (Gluc+), cells were pre-cultured in LB containing arabinose, pelleted, washed twice with 0.85% saline solution, diluted into fresh LB containing glucose to an OD_600_ less than 0.001 and then cultured until mid exponential phase (OD_600_ 0.5). For intermediate depletion of YbeY (indicated as Ara+/Gluc+), cells were grown in LB containing arabinose until early exponential phase (OD_600_ 0.1), pelleted, washed twice and subsequently grown in LB containing glucose until mid exponential phase.

To analyze growth, C6706 Wt pY and *ΔybeY* pY strains were cultured as described above and growth was monitored for 8–10 h until saturation. Additionally, the *ΔybeY* pY mutant strain was grown in the absence of arabinose (Ara-) for depletion of YbeY and subsequently subcultured a second time into LB medium lacking arabinose (Ara-/Ara-) to further deplete YbeY.

Antibiotics were used at the following concentrations: kanamycin (Kan: 40 µg/ml), tetracycline (Tet: 0.05 µg/ml), gentamycin (Gen: 2.0 µg/ml for *V. cholerae* and 0.4 µg/ml for *E. coli*), kasugamycin (Kas: 100 µg/ml), chloramphenicol (Cm: 0.25 µg/ml *V. cholerae* and 2.5 µg/ml *E. coli*), erythromycin (Ery: 0.5 µg/ml), rifampicin (Rif: 0.1 µg/ml), ampicillin (Amp: 2 µg/ml), and cefotaxime (Cef: 0.0002 µg/ml for *V. cholerae* and 0.2 µg/ml for *E. coli*).

### Ribosome isolation and profiles

70 S ribosomes, and 50 S and 30 S subunits from *V. cholerae* C6706, *V. cholerae ΔybeY* pY mutant, *V. cholerae rng*::Tn mutant, *V. cholerae rne-CTD*::Tn mutant, *V. cholerae rnr*::Tn mutant, *E. coli* MC4100, and *E. coli ΔybeY* mutant were isolated as described previously [93] with minor modifications. Briefly, log phase cultures (OD_600_ 0.5) grown in LB at 37°C were harvested, resuspended in ice-cold buffer A (20 mM HEPES pH 7.5, 50 mM NH_4_Cl, 10 mM MgCl_2_, 5 mM β-mercaptoethanol, 0.1 mM PMSF), and lysed by French press (two passes at 11,000 psi). All subsequent steps were carried out at 4°C. Cell debris was removed by low speed centrifugation. The 30 S supernatant was obtained by two spins at 30,000 g for 30 min and was subsequently subjected to centrifugation at 100,000 g for 3 h. The final pellet containing the ribosomes was resuspended in buffer A and loaded onto a 10–40% sucrose gradient made in buffer A. After centrifugation at 150,000 g for 7 h, the 70 S ribosomes and ribosomal subunits were collected by monitoring the fractions spectro-photometrically (A_260_).

### Isolation and analysis of RNA

Total RNA from log phase cultures (OD_600_ 0.5) grown in LB at 37°C and rRNA from purified ribosome fractions were extracted using TRIzol (Ambion) according to the manufacturer's protocol. Samples used for RT-qPCR and sRNA analysis were treated with TURBO DNase (Ambion) to remove any DNA contamination. Total rRNA profiles were analyzed by Synergel/agarose gel electrophoresis as described [94].

### cDNA synthesis and RT-qPCR

cDNA was synthesized using the iScript cDNA Synthesis Kit (Biorad) according to the manufacturer's protocol, and 1 µg of total RNA per 20 µl reaction. Relative expression levels were determined by RT-qPCR using the LightCycler 480 Real-time PCR system. Primers were designed using Primer Express 3.0 (optimal primer length of 20 bases, optimal amplicon length of 100 bases, GC-content of 50% and Tm of 58–60°C, see Table S1). 10 ng of cDNA and 0.2 µM of each primer was mixed with LightCycler 480 Cyber Green I master mix in a 20 µl reaction. PCR conditions were: a pre-incubation of 5 min at 95°C, an amplification stage of 45 cycles of 20 s at 95°C, 30 s at 57°C and 20 s at 72°C, and a melting curve stage of 5 s at 95°C, 1 min at 65°C increased to 97°C with steps of 2.2°C/s. All reactions were performed in triplicate and samples without reverse transcriptase were used as a control to assess genomic DNA contamination. The raw data was analyzed using LightCycler 480 Software (1.5.0 SP3), normalized against 5 S rRNA and the wild-type strain C6706 (without the pY maintenance plasmid; without additional carbon source) was used as calibrator condition. Relative gene expression was calculated using the Livak method [95].

### Northern blot analysis of RNA

RNA was separated by denaturing polyacrylamide gel electrophoresis (6% polyacrylamide/7 M urea). The transfer of RNA onto Nytran membrane and Northern blot hybridization were as described in [96] with minor modifications. DNA oligonucleotides were endlabeled with ^32^P using T4 polynucleotide kinase (New England Biolabs). Prehybridizations were carried out at 42°C and hybridizations were carried out at 40°C–45°C. Following hybridization, membranes were washed at room temperature with 6×–2× SSC. Autoradiograms were analyzed by phosphorimager using a Typhoon scanner and ImageQuant software.

### Mapping of 5′ and 3′ termini of rRNA

To map the 5′ termini of rRNA, primer extension assays were performed using Superscript II reverse transcriptase (Invitrogen). Typically, a 10 µl reaction contained 100 ng of total RNA or 25–50 ng of rRNA isolated from ribosomes, 100,000 cpm of the ^32^P-labeled DNA oligonucleotide and 2 µl of the 5× reaction buffer (Invitrogen). After denaturation (65°C, 5 min), samples were chilled on ice for 5 min and then brought to room temperature for 5 min. 0.5 µl of DTT solution (Invitrogen), 0.25 µl of a 10 mM dNTP solution and 50 U of Superscript II (Invitrogen) were added. The reverse transcription reaction was carried out at 45°C for 20 min. The reaction was stopped by addition of loading dye containing formamide and heat denaturation. Reaction products were separated on denaturing polyacrylamide gels (6–10% polyacrylamide/7 M urea).

The 3′ termini of rRNA was mapped by site-specific RNase H cleavage assay as described by Deutscher and colleagues [29], [97], [98] with minor modifications, followed by Northern hybridization using probes specific for the mature 3′ termini of 16 S rRNA, 23 S rRNA or 5 S rRNA, respectively. Briefly, 100 ng of total RNA or 50 ng of rRNA isolated from ribosomes were mixed with a chimeric RNA/DNA oligonucleotide and 1 µl of 10× RNase H buffer (New England Biolabs) in a 10 µl reaction. After denaturation (95°C, 1 min), samples were brought to 65°C for 5 min, then 55°C for 15 min, 35°C for 15 min and then to room temperature for another 15 min to allow for formation of the DNA:RNA hybrid. 2.5 U of RNase H (New England Biolabs) was added. Samples were incubated for 60 min at 37°C. The reaction was stopped by addition of loading dye containing formamide and heat denaturation. Reaction products were separated on denaturing polyacrylamide gels (6–10% polyacrylamide/7 M urea) and analyzed by Northern blot hybridization.

### Overexpression and purification of YbeY and RNase R

To construct the overexpression plasmid, *ybeY* of *E. coli* (b0659) and *V. cholerae* (VC0960) was cloned into pET28a under control of a T7 promoter with an N-terminal His-MBP-TEV(site)-tag. Purification of YbeY without RNase I contamination was accomplished by expression in BL21 (DE3) *Δrna* [18]. To improve activity and yield, pET28a_YbeY was co-transformed with pGro7, a chaperone plasmid that encodes both GroES and GroEL under an arabinose inducible promoter [99]. MBP-YbeY was purified using amylose resin (New England Biolabs), the MBP-tag was cleaved by Turbo TEV protease (Eton Bioscience Inc.) and removed by size exclusion chromatography as described previously [18]. The purified YbeY protein was subjected to mass spectrometry to assess its purity.

*rnr* (encoding RNase R; VC0960) of *V. cholerae* was cloned into pET15b under control of a T7 promoter with an N-terminal His-tag. After overexpression in BL21 (DE3) *Δrna* [18], RNase R was purified by affinity chromatography using Talon resin (Clontech) following the manufacturer's protocol for batch-gravity-flow purification of proteins.

### *In vitro* RNase assays

All YbeY RNase assays were carried out as described previously in 50 mM HEPES pH 7.5 in a 20 µl volume [18]. Total RNA (250 ng) was incubated with *E. coli* or *V. cholerae* YbeY (5–10 µM) for 1 h at 37°C. Digestion products were separated on Synergel/agarose gels. Short synthetic RNA substrates were labeled at their 5′ terminus using γ-^32^P-ATP and T4 polynucleotide kinase (New England Biolabs). For each reaction, 0.03 µM short RNA substrate was incubated with *V. cholerae* or *E. coli* YbeY (1–5 µM) for 1 h at 37°C. Products were separated on a 10% denaturing polyacrylamide gel containing 7 M urea.

70 S ribosomes (0.05 µM) isolated from *V. cholerae* Δ*ybeY* pY (grown under maximum YbeY depletion) and *V. cholerae* C6706 Wt pY (grown in the presence of arabinose) were incubated with YbeY and RNase R of *V. cholerae* (0.1 µM) or *E. coli* (0.05 µM), either alone or together in 50 mM HEPES pH 7.5 and 10 mM MgCl_2_ for 1 h at 37°C. Ribosomal RNA was subsequently isolated using TRIzol, separated by Synergel/agarose gel electrophoresis and stained with ethidium bromide.

### Mouse infections

Infant mice were inoculated with *V. cholerae ΔybeY* pY cells grown in the absence or presence of 0.2% arabinose. We assessed their ability to colonize the mouse intestine in competition with the parental strain *V. cholerae* C6706 as previously described [100].

### Western blot analysis of Cholera toxin

The levels of cholera toxin produced by *V. cholerae* C6706 Wt pY and *ΔybeY* pY mutant were analyzed by western blotting. Supernatants of cultures (10 mL) grown at 37°C in LB medium in the presence of 0.1% arabinose or 0.2% glucose as indicated, were collected after 8 h by centrifugation and subsequently passed through a 0.2 µm cellulose acetate filter (VWR). The total protein content of the samples was estimated by Bradford using BSA as reference. 5 ng of supernatant protein mixed with 5 µg of BSA were applied to a 12% SDS-PAGE and transferred onto an Immobilon-P membrane (PVDF) following standard procedures [87]. Non-specific sites were blocked using 5% milk powder in TBS-T (0.05% Tween20 in 20 mM Tris-HCl, 150 mM NaCl, pH 7.5) by incubation at room temperature for 4 h. The primary Rabbit polyclonal anti-Cholera toxin antibody (Sigma-Aldrich) was dissolved in 5% milk powder/TBS-T at a concentration of 0.05 µg/ml and incubated with the membrane overnight at 4°C. After 5 washes with TBS-T for 5 min, the secondary antibody (Anti-Rabbit IgG HRP; Thermo Scientific) was dissolved in 5% milk/TBS-T (0.002 µg/ml) and incubated with the membrane for 2 h at room temperature. After 5 washes with TBS-T for 5 min, 1 ml of SuperSignal West Dura Chemiluminescent Substrate (Thermo Scientific) was added for 5 min before exposing the X-ray film.

### Stress tests and complementation assays

Stress sensitivity of the *V. cholerae ΔybeY* pY mutant compared to the C6706 Wt pY was determined by spotting serially diluted (1∶10, starting at OD_600_ 0.1) cultures, grown overnight in LB medium and subsequently washed with 0.85% saline solution, onto LB plates containing various antibiotics or 0.1 mM H_2_O_2_ along with either 0.1% arabinose or 0.2% glucose as indicated. For UV survival, cultures of the *V. cholerae* strains were spotted onto LB plates and irradiated with a UV dose of 20 J/m^2^ using a G15T8 UV lamp (GE) at 254 nm, then incubated in the dark. To determine heat sensitivity of C6706 Wt pY and *ΔybeY* pY, cells from an early exponential culture supplemented with 0.2% glucose were incubated for 3 h at 37°C and 45°C before spotting as a dilution series onto LB plates supplemented with 0.2% glucose; plates were subsequently incubated for 18 h at 37°C. Cold sensitivity of C6706 Wt pY and *ΔybeY* pY mutant cells was resolved by spotting serially diluted cultures of a late exponential culture, grown in the presence of 0.1% arabinose, onto LB plates containing 0.2% glucose; plates were incubated for 2 days at 37°C and 18°C as indicated.

Similarly, complementation of the *E. coli ΔybeY* mutant by *ybeY* of various pathogens was determined by spotting serially diluted cultures (1∶10, starting at OD_600_ 0.1), grown overnight in LB medium, onto LB plates containing various antibiotics or 0.75 mM H_2_O_2_. A higher UV dose of 60 J/m^2^ was used to measure UV resistance of the *E. coli* strains. Heat sensitivity of *E. coli* cells was determined by spotting serially diluted cultures of a late exponential culture onto LB plates; plates were incubated for 18 h at 45°C as indicated. The complementation vectors constitutively express *ybeY* of *E. coli* MC4100 (pY_Ec), *V. cholerae* N16961 (pY_Vc), *P. aeruginosa* H37Rv (pY_Pa), *M. tuberculosis* H37v (pY_Mt), or *S. pneumoniae* R6 (pY_Sp).

Bile-salt sensitivity of C6706 Wt pY and *ΔybeY* pY mutant was determined by growth in LB medium supplemented with 2.0% bile salt and 0.1% arabinose as specified. The number of colony forming units (CFU) was determined after 0, 1, 2, and 3 h of bile-salt treatment.

Pigment formation of C6706 Wt pY and *ΔybeY* pY mutant was determined by spinning down a similar number of cells grown to mid-log phase in LB medium containing 0.1% arabinose or 0.2% glucose as indicated.

## Supporting Information

Figure S1 **Growth analysis of** ***V. cholerae*** **Δ** ***ybeY*** **.** Growth curve of C6706 Wt pY and the Δ*ybeY* pY strain in LB medium at 37°C. The Wt and mutant strains were grown in medium supplemented with arabinose (Ara+). The Δ*ybeY* pY strain was diluted into medium lacking arabinose (Ara-) for depletion of YbeY, grown to saturation and then subcultured a second time into fresh medium lacking arabinose (Ara-/Ara-).
(TIF)

Figure S2 **Analysis of 23 S rRNA and 5 S rRNA in** ***V. cholerae*** **Δ** ***ybeY*** **.** Mapping of 5′ and 3′ termini of A) 23 rRNA and B) 5 S rRNA from C6706 Wt pY and the Δ*ybeY* pY strain. “P” and “M” specify the positions of bands derived from the precursor and mature forms of 23 S rRNA and 5 S rRNA. “pY” indicates that *ybeY* is expressed from a plasmid. Ara+, cells were grown in LB in the presence of arabinose. Gluc+, cells were grown in LB in the presence of glucose. Ara+/Gluc+, intermediate YbeY depletion by switching the carbon source of the Δ*ybeY* pY strain in early exponential phase from arabinose to glucose (for details see Materials and Methods).
(TIF)

Figure S3 **Analysis of** ***V. cholerae*** **YbeY's** ***in vitro*** **RNase activity.** **A**) Total RNA (250 ng) isolated from *V. cholerae* can be degraded by purified YbeY of *V. cholerae* (10 µM), similar to *E. coli* YbeY (5 µM). EDTA (50 mM) inhibits YbeY's RNase activity. RNA was analyzed by agarose gel electrophoresis. The positions of the 23 S and 16 S rRNA are indicated. **B–C**) Purified YbeY of *V. cholerae* (1–5 µM) shows endoribonuclease activity on 5′ ^32^P-labled oligoribonucleotides (0.03 µM): hairpin substrate in **B**) and ssRNA in **C**). Digestion products were analyzed by polyacrylamide gel electrophoresis. **D**) *In vitro* ribosome quality control by *V. cholerae* (*Vc*) and *E. coli* (*Ec*) YbeY together with RNase R. 70 S ribosomes of C6706 Wt pY grown in the presence of arabinose (Ara+) were incubated with YbeY, RNase R, or a mixture of YbeY and RNase R from *E. coli* or *V. cholerae* as indicated. The positions of 23 S and 16 S rRNAs are indicated based on their mobility.
(TIF)

Figure S4 **Ribosome profiles of** ***V. cholerae*** **mutant strains with transposon insertions in genes encoding RNase E (** ***rne*** **-CTD::Tn), RNase G (** ***rng*** **::Tn) or RNase R (** ***rnr*** **::Tn).** Analysis of ribosome profiles in C6706 Wt pY (W), Δ*ybeY* pY (Δ), *rne-CTD::Tn* (E), *rng::Tn* (G) and *rnr::Tn* (R). Wt pY and Δ*ybeY* pY cells were first grown in LB medium supplemented with arabinose and then subcultured into glucose-containing medium for depletion of YbeY; all other strains were grown in LB medium without additional carbon source. Polysomes, 70 S, 50 S and 30 S ribosomes are indicated.
(TIF)

Figure S5 **YbeY is highly conserved among bacteria.** Sequence alignment of YbeY proteins from *E. coli* MG1655 (Ec), *V. cholerae* N16961 (Vc), *P. aeruginosa* PAO1 (Pa), *M. tuberculosis* H37Rv (Mt), and *S. pneumoniae* R6 (Sp). The highly conserved H3XH5XH motive in the catalytic pocket is indicated.
(TIF)

Table S1 **Oligonucleotides used in this study.**
(PDF)
