# Supplementary figures and images for: The Highly Conserved Bacterial RNase YbeY Is Essential in Vibrio cholerae, Playing a Critical Role in Virulence, Stress Regulation, and RNA Processing
Source: PLoS Pathog. 2014 Jun 5;10(6):e1004175. doi: 10.1371/journal.ppat.1004175 (PMC4047096; doi:10.1371/journal.ppat.1004175)

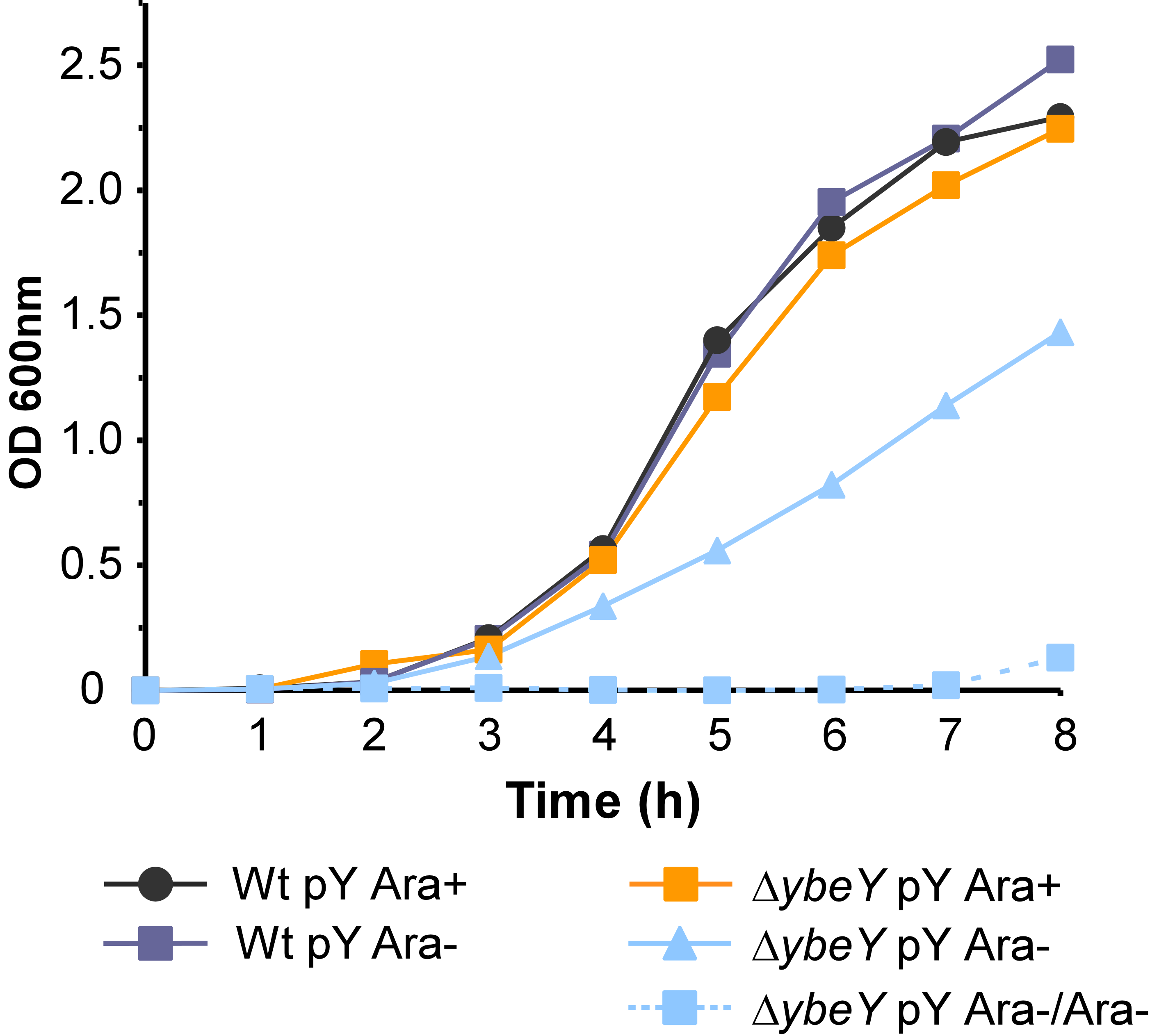

Supplement: Figure S1 — Growth analysis of V. cholerae Δ ybeY . Growth curve of C6706 Wt pY and the ΔybeY pY strain in LB medium at 37°C. The Wt and mutant strains were grown in medium supplemented with arabinose (Ara+). The ΔybeY pY strain was diluted into medium lacking arabinose (Ara-) for depletion of YbeY, grown to saturation and then subcultured a second time into fresh medium lacking arabinose (Ara-/Ara-). (TIF) [file ppat.1004175.s001.tif]

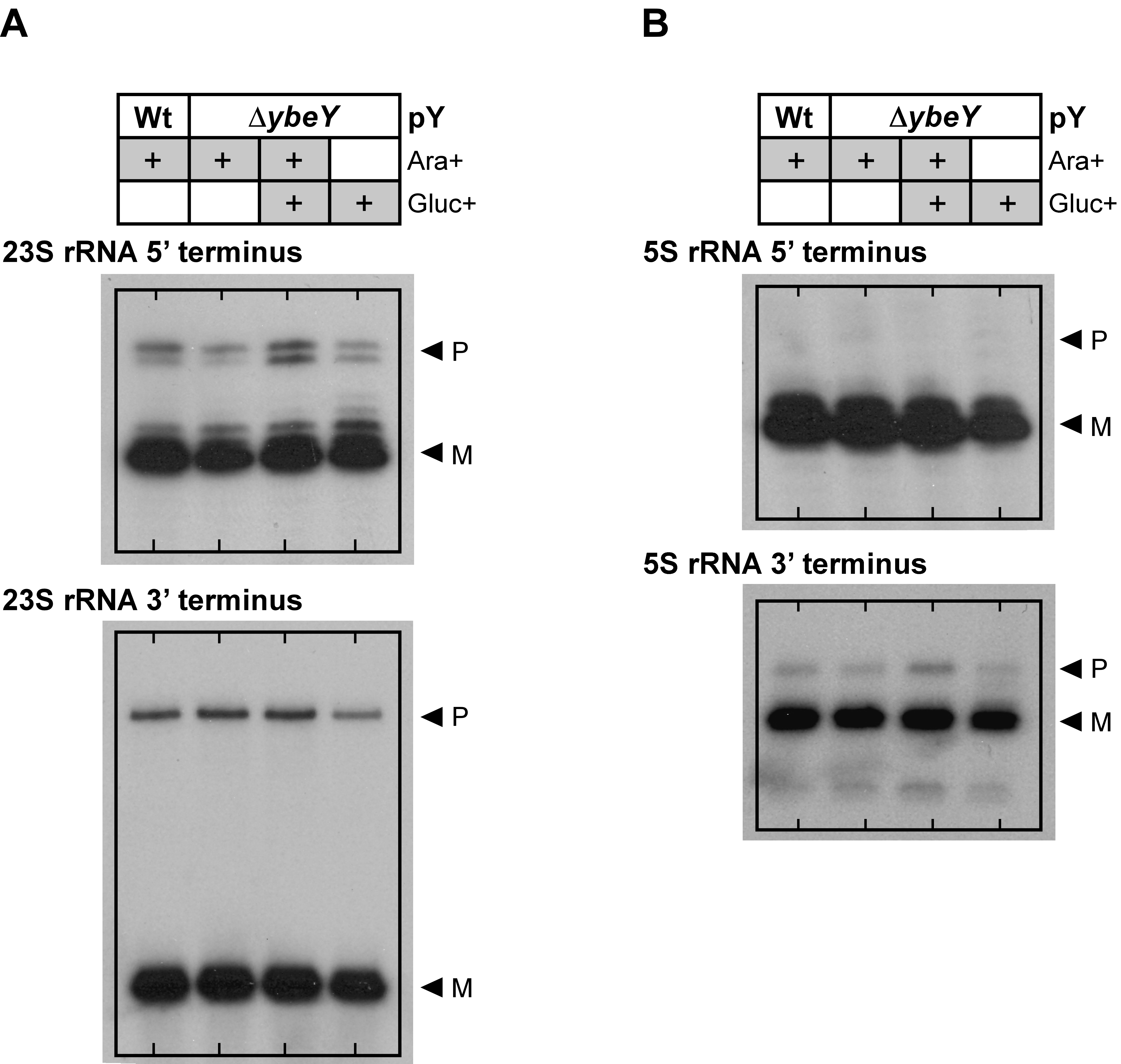

Supplement: Figure S2 — Analysis of 23 S rRNA and 5 S rRNA in V. cholerae Δ ybeY . Mapping of 5′ and 3′ termini of A) 23 rRNA and B) 5 S rRNA from C6706 Wt pY and the ΔybeY pY strain. “P” and “M” specify the positions of bands derived from the precursor and mature forms of 23 S rRNA and 5 S rRNA. “pY” indicates that ybeY is expressed from a plasmid. Ara+, cells were grown in LB in the presence of arabinose. Gluc+, cells were grown in LB in the presence of glucose. Ara+/Gluc+, intermediate YbeY depletion by switching the carbon source of the ΔybeY pY strain in early exponential phase from arabinose to glucose (for details see Materials and Methods). (TIF) [file ppat.1004175.s002.tif]

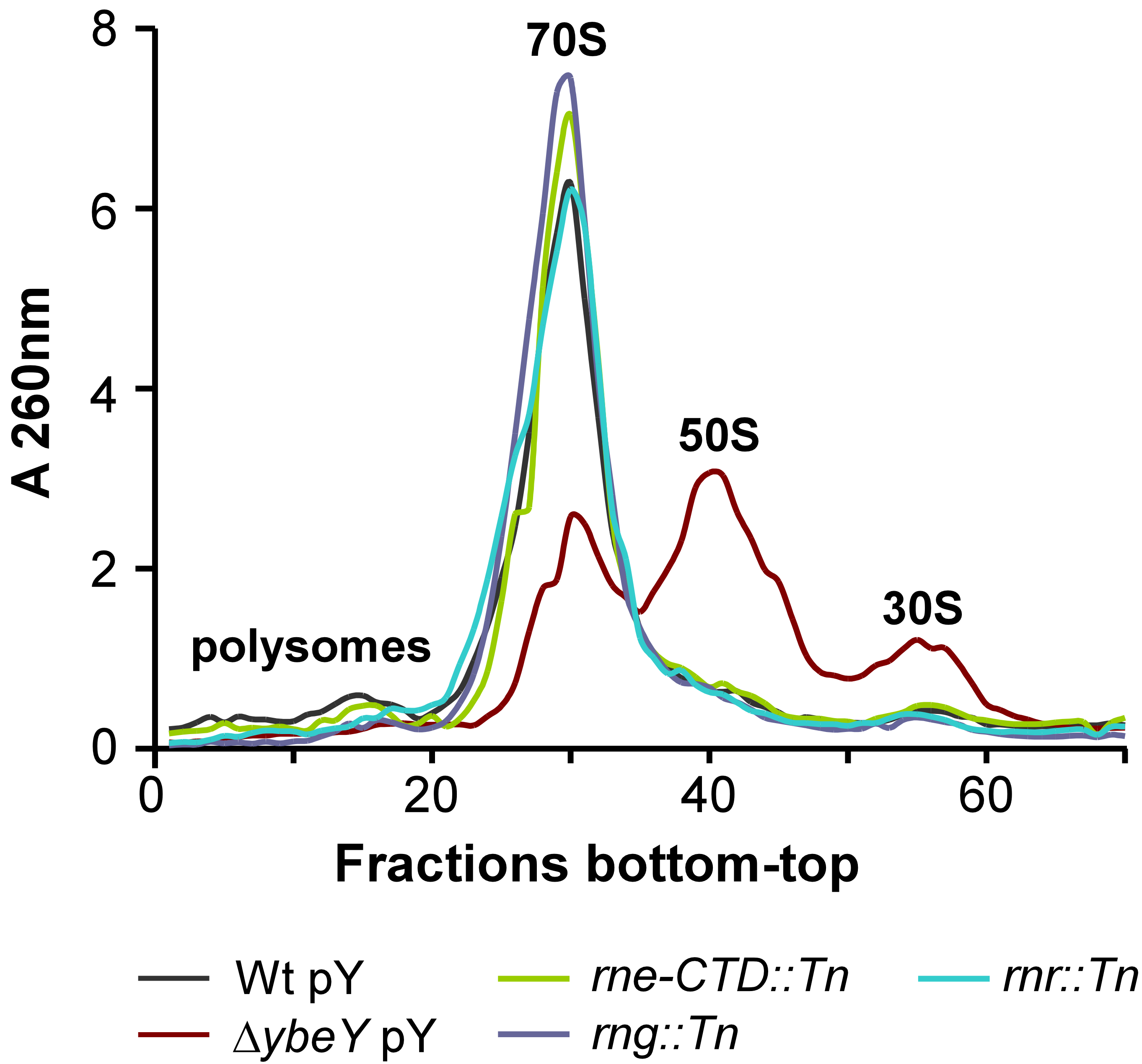

Supplement: Figure S4 — Ribosome profiles of V. cholerae mutant strains with transposon insertions in genes encoding RNase E ( rne -CTD::Tn), RNase G ( rng ::Tn) or RNase R ( rnr ::Tn). Analysis of ribosome profiles in C6706 Wt pY (W), ΔybeY pY (Δ), rne-CTD::Tn (E), rng::Tn (G) and rnr::Tn (R). Wt pY and ΔybeY pY cells were first grown in LB medium supplemented with arabinose and then subcultured into glucose-containing medium for depletion of YbeY; all other strains were grown in LB medium without additional carbon source. Polysomes, 70 S, 50 S and 30 S ribosomes are indicated. (TIF) [file ppat.1004175.s004.tif]

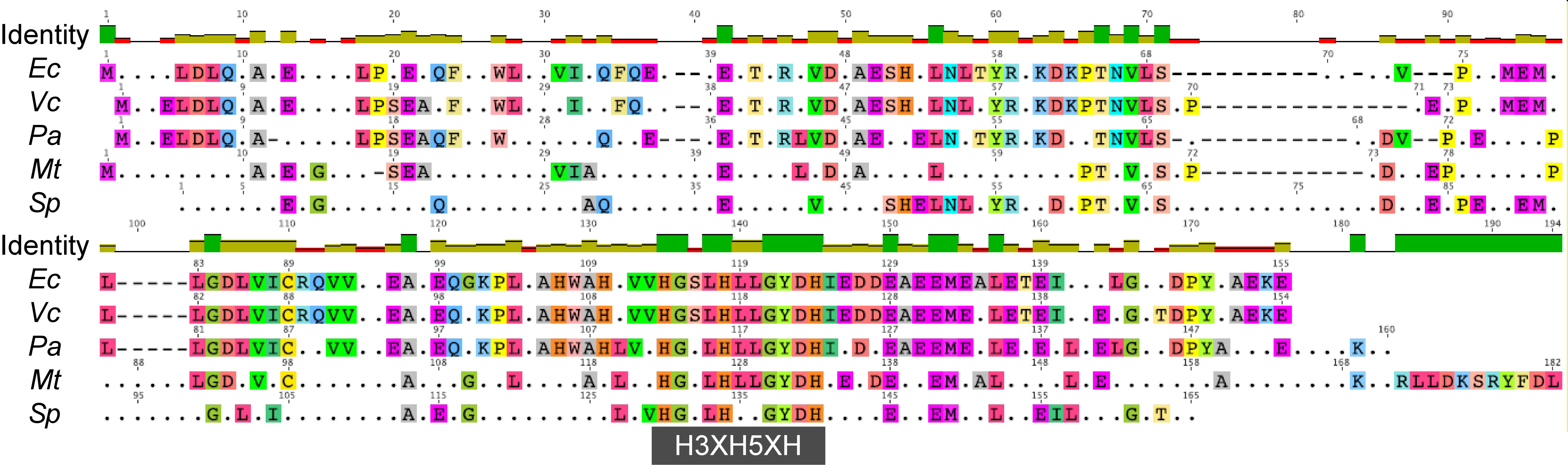

Supplement: Figure S5 — YbeY is highly conserved among bacteria. Sequence alignment of YbeY proteins from E. coli MG1655 (Ec), V. cholerae N16961 (Vc), P. aeruginosa PAO1 (Pa), M. tuberculosis H37Rv (Mt), and S. pneumoniae R6 (Sp). The highly conserved H3XH5XH motive in the catalytic pocket is indicated. (TIF) [file ppat.1004175.s005.tif]
