# Supplementary material for: The Highly Conserved Bacterial RNase YbeY Is Essential in Vibrio cholerae, Playing a Critical Role in Virulence, Stress Regulation, and RNA Processing
Source: PLoS Pathog. 2014 Jun 5;10(6):e1004175. doi: 10.1371/journal.ppat.1004175 (PMC4047096; doi:10.1371/journal.ppat.1004175)
Supplement: Table S1 — Oligonucleotides used in this study. (PDF) [file ppat.1004175.s006.pdf]

Supplemental Table S1.

rRNA: mapping and hybridization

name:	sequence:	used for:
Vc/Ec_16S_5PE	5’-GCCTGCCGCCAGCGTTCAAT-3’	5’ mapping of *V. cholerae* and *E. coli* 16S rRNA
Vc/Ec_23S_5PE	5’-GTCCTTCATCGCCTCTGACTGCC-3’	5’ mapping of *V. cholerae* and *E. coli* 23S rRNA
Vc_5S_5PE	5’-TGGAATCAGGTGGGTCCAAAACGCT-3’	5’ mapping of *V. cholerae* 5S rRNA
Ec_5S_5PE	5’-TGGGGTCAGGTGGGACCACCGCGCT-3’	5’ mapping of *E. coli* 5S rRNA
Vc_16S_3’CHIM	5’-mCmCCGAAmGmGmUmUmAmAmAmCmUmAmCmCmU-3’	RNA/DNA chimera for 3’ mapping of *V. cholerae* 16S rRNA
Ec_16S_3’CHIM	5’-mCmCCGAAmGmGmUmUmAmAmGmCmUmAmCmCmU-3’	RNA/DNA chimera for 3’ mapping of *E. coli* 16S rRNA
Vc_23S_3’CHIM	5’-mAmACGCTmUmAmCmAmCmAmCmCmCmUmGmCmC-3’	RNA/DNA chimera for 3’ mapping of *V. cholerae* 23S rRNA
Vc_5S_3’CHIM	5’-mAmCGCTGmUmUmUmCmGmUmUmUmCmAmCmUmUmCmUmGmAmG-3’	RNA/DNA chimera for 3’ mapping of *V. cholerae* 5S rRNA
Vc_16S_3’	5’-AAGGAGGTGATCCAGCGCCA-3’	hybridization probe for 3’ mapping of *V. cholerae* 16S rRNA
Ec_16S_3’	5’-TAAGGAGGTGATCCAACCGCA-3’	hybridization probe for 3’ mapping of *E. coli* 16S rRNA
Vc_23S_3’	5’-GGTTAAGCCTCACGGGCAATTAGTA-3’	hybridization probe for 3’ mapping of *V. cholerae* 23S rRNA
Vc_5S_3’	5’-GCCTGGCGATGTTCTACTCTCACAT-3’	hybridization probe for 3’ mapping of *V. cholerae* 5S rRNA
mN … 2'-*O*-methyl RNA bases; N … standard DNA bases		

sRNA: mapping and hybridization

name:	sequence:	used for detection of:
Vc_micX	5’-AAAAACTGTTAGTCGTGAACTAGTAAGGA-3’	*V. cholerae* micX sRNA
Vc_vrrA	5’-ACACAAGTATAGGAGTTAATAAAAAGGAAAAAGCAGC-3’	*V. cholerae* vrrA sRNA
Vc_tarB_1	5’-AACAATACATGAGGTAACCGCTCAT-3’	*V. cholerae* tarB sRNA
Vc_tarB_2	5'-GCCAAAAAGTGCTTAATCGTCAGCTTGTAA-3'	*V. cholerae* tarB sRNA
Vc_6S_1	5’-CGCTGACGAACACCCCAGGGTAAAT-3’	*V. cholerae* 6S

		sRNA
Vc_6S_2	5'-CCCTGTTATGTTGCTTATCGGCTCA-3'	*V. cholerae* 6S sRNA
Vc_qrr1-4	5'- ACAACGTCAGTTGGCTAGGTGACCC -3'	*V. cholerae* qrr1-4 sRNAs

RT-qPCR

name:	sequence:	used for quantification of *V. cholerae* genes:
YbeYqPCRFw	5'-TGCTTTGTGGCTCACTCGAA-3'	*ybeY* (forward)
YbeYqPCRRv	5'-GTTCAAATTCAGCGCATGGC-3'	*ybeY* (reverse)
5SqPCRFw	5'-TAGCGTTTTGGACCCACCTG-3'	5S rRNA (forward)
5SqPCRRv	5'-GACCCCACACTACCATCGAC-3'	5S rRNA (reverse)
RneNTqPCRFw	5'-TGCGTGAAGGTCAGGAAGTC-3'	*rne* N-terminal domain (forward)
RneNTqPCRRv	5'-AGATAGCTACCCGCCAGACT-3'	*rne* N-terminal domain (reverse)
RneCTqPCRFw	5'-CTTCTTCGGCGCACTGAAAG-3'	*rne* C-terminal domain (forward)
RneCTqPCRRv	5'-CTGTTGACGTTCCGGTTTCG-3'	*rne* C-terminal domain (reverse)
RnrVcqPCRFw	5'-AACCCACAAGTCGATCGCTT-3'	*rnr* (forward)
RnrVcqPCRRv	5'-GCGAGTTCATTACCGCTTCG-3'	*rnr* (reverse)
